# Molecular characterization, targeting and expression analysis of chloroplast and mitochondrion protein import components in *Nicotiana benthamiana*

**DOI:** 10.3389/fpls.2022.1040688

**Published:** 2022-10-26

**Authors:** María Sáiz-Bonilla, Andrea Martín Merchán, Vicente Pallás, Jose Antonio Navarro

**Affiliations:** Laboratory of Plant Molecular Virology, Department of Molecular and Evolutionary Plant Virology, Institute for Plant Molecular and Cell Biology, Consejo Superior de Investigaciones Científicas-Universitat Politècnica de València, Valencia, Spain

**Keywords:** nicotiana benthamiana, chloroplasts, mitochondria, translocon receptor, Toc, Tom, protein transport

## Abstract

Improved bioinformatics tools for annotating gene function are becoming increasingly available, but such information must be considered theoretical until further experimental evidence proves it. In the work reported here, the genes for the main components of the translocons of the outer membrane of chloroplasts (Toc) and mitochondria (Tom), including preprotein receptors and protein-conducting channels of *N. benthamiana*, were identified. Sequence identity searches and phylogenetic relationships with functionally annotated sequences such as those of *A. thaliana* revealed that *N. benthamiana* orthologs mainly exist as recently duplicated loci. Only a Toc34 ortholog was found (NbToc34), while Toc159 receptor family was composed of four orthologs but somewhat different from those of *A. thaliana*. Except for NbToc90, the rest (NbToc120, NbToc159A and NbToc159B) had a molecular weight of about 150 kDa and an acidic domain similar in length. Only two orthologs of the Tom20 receptors, NbTom20-1 and NbTom20-2, were found. The number of the Toc and Tom receptor isoforms in *N. benthamiana* was comparable to that previously reported in tomato and what we found in BLAST searches in other species in the genera *Nicotiana* and *Solanum*. After cloning, the subcellular localization of *N. benthamiana* orthologs was studied, resulting to be identical to that of *A. thaliana* receptors. Phenotype analysis after silencing together with relative expression analysis in roots, stems and leaves revealed that, except for the Toc and Tom channel-forming components (NbToc75 and NbTom40) and NbToc34, functional redundancy could be observed either among Toc159 or mitochondrial receptors. Finally, heterodimer formation between NbToc34 and the NbToc159 family receptors was confirmed by two alternative techniques indicating that different Toc complexes could be assembled. Additional work needs to be addressed to know if this results in a functional specialization of each Toc complex.

## Introduction

Over time, the vast majority of the genes (93–99%) from the prokaryotic ancestors of the mitochondria and plastids in plants were lost or/and transferred to the nucleus. Currently, mitochondria and chloroplasts contain close to a thousand or several thousand proteins, respectively, but at best, their remnant endosymbiont genomes encode only about a hundred of them. These numbers imply that encoding organelle-destined proteins must be transported back into the respective organelle after their synthesis in the cytosolic ribosomes (Bykov et al., 2020; Rochaix, 2022). In addition, the majority of the oligomeric protein complexes found in mitochondria and plastids, such as ribosomes, electron transport chains, or even the most abundant enzyme in the world, the ribulose-1,5-bisphosphate carboxylase/oxygenase, consist of subunits of dual genetic origin (Priesnitz and Becker, 2018; Ramundo et al., 2020). A tightly nuclear-organelle coordinated mechanism must exist to maintain organelle function and biogenesis in these circumstances. This system must ensure the availability of the different subunits encoded on separated and compartmentalized genomes in the correct stoichiometry for complex assembly and their appropriate transport at the time and place required. The latter process was highly dependent on the successful acquirement of an organellar protein import apparatus that, despite mitochondria appeared much earlier than plastids during eukaryotic cell evolution, arose *de novo* in both organelles. Although mitochondrion and chloroplast transport systems do not share any homology, they are mechanistically similar (Peeters and Small, 2001; Kunze and Berger, 2015).

Reciprocal crosstalk between the nucleus and both mitochondria and chloroplasts is necessary to coordinate their respective gene expression and ensure the appropriate synthesis of protein working in common complexes during biogenesis and plant growth (Woodson and Chory, 2008). Nevertheless, mitochondria and chloroplasts are not only organelles primarily devoted to energy conversion. Their involvement in sensing environmentally stressful conditions, cell death and redox signaling is currently more than evident and known to be related to their physiological state (Kleine and Leister, 2016; Wang et al., 2020). In response to changes in current functions, mitochondria and chloroplasts can coordinately initiate a signaling cascade, known as the retrograde response pathway, to modulate the expression of nuclear genes (Wang et al., 2020). Therefore, these organelles have become logical targets for pathogen effectors. Small fungus effectors target mitochondria and chloroplasts to suppress the hypersensitive response (Tzelepis et al., 2021), and the localization of the coat protein of cucumber necrosis virus into the chloroplast stroma attenuates host defense response (Alam et al., 2021). More recently, we have also shown that the mitochondrial and chloroplast dual targeting of melon necrotic spot virus coat protein modulates chloroplast-to-nucleus communication, mitigating tissue necrosis and favoring the local and systemic spread of the infection (Navarro et al., 2021).

Mitochondria and chloroplasts are surrounded by a double-membrane that defines an intermembrane space and an innermost subcompartment termed stroma in chloroplasts or matrix in mitochondria. In addition, chloroplast stroma holds a third membrane system called thylakoids. It consists of a set of interconnected and highly specialized stacked membrane sacs where many essential photosynthesis-related proteins are located. Sorting newly synthesized proteins and their import into the correct organelle and/or intraorganellar compartment requires the presence of specific targeting signals mostly located at the N-terminus in the case of the mitochondrial matrix and stromal proteins, the well-known transit peptide (TP) and presequence, respectively. Although internal or C-terminal targeting signals exist, they are mainly found in proteins destined for organelle membranes or intermembrane spaces (Shi and Theg, 2013; Murcha et al., 2014; Kunze and Berger, 2015). The targeting of precursor proteins from the cytosol to mitochondria or chloroplasts also requires the help of some chaperones, especially members of the Hsp70/90 families, and, specifically for chloroplast import, several cytosolic factors belonging to the 14-3-3 family of phosphoserine binding proteins. Both are thought to maintain precursor proteins in an unfolded, transport-competent state (May and Soll, 2000; Voos and Röttgers, 2002; Flores-Pérez and Jarvis, 2013). Once inside the organelle, targeting signals of these preproteins can undergo or not a specific cleavage giving rise to mature proteins (Teixeira and Glaser, 2013). The main chloroplast or mitochondrion gateway is a proteinaceous channel that, together with several membrane-associated receptors, forms a complex translocon protein machinery called Toc (translocase of the outer chloroplast membrane) or Tom (translocase of the outer mitochondrial membrane). Soluble proteins further destined for chloroplast stroma and mitochondrial matrix pass across the inner membranes through a second translocon known as Tic (translocon of the inner chloroplast membrane) and Tim17:23 (translocon of the inner mitochondrial membrane 17:23), respectively (Murcha et al., 2014; Richardson et al., 2014).

The central core of the Toc complex is composed of two GTP-regulated receptors, Toc34 and Toc159, that bind to the TPs of preproteins and initiate the protein import through Toc75, a β-barrel membrane channel (Eckart et al., 2002; Hinnah et al., 2002). In vascular plants, Toc34 and Toc159 exist in multiple isoforms, allowing them to form structurally different Toc complexes. Thus, the import of a specific subset of proteins could be driven by each Toc complex in response to environmental factors and/or the developmental and physiological state (Ivanova et al., 2004; Kubis et al., 2004; Dutta et al., 2014; Wiesemann et al., 2019). The membrane topology of Toc75 and, especially, the orientation of its soluble N-terminal polypeptide transport-associated (POTRA) domains have been a matter of debate during the last decade since they were reported by different researchers either facing the cytoplasm or chloroplast intermembrane space. The final model of protein translocation may differ depending on POTRA orientation: in the intermembrane space, POTRA domains and Tic22 are suggested to act as chaperones that facilitate preprotein transfer to the Tic complex (Kasmati et al., 2013; Gross et al., 2020); alternatively, a cytoplasmic exposure of the POTRA domains, which provides a Toc33 binding site, could regulate the GTPase activity of the TOC receptors (Sommer et al., 2011).

More than five protein import pathways operate in plant mitochondria depending on protein topology, but all of them employ the Tom complex to a greater or lesser extent (Bausewein et al., 2020). The core subunit of the Tom complex is the transmembrane β-barrel protein Tom40 (Rapaport and Neupert, 1999; Hu et al., 2019), while precursor protein recognition with presequences generally occurs *via* the family of the Tom20 receptors. In addition, plant-specific receptor Om64 (outer membrane 64) is a paralogue of Toc64 that could play a role analogous to Tom71 and Tom70 in yeast and animals, respectively, in cytosolic chaperone binding and insertion of hydrophobic and multispanning α-helical proteins of the outer membrane (Lister et al., 2007; Murcha et al., 2014).

Concerning resident proteins of chloroplasts and mitochondria in green plants and except for a few examples, such as *Pisum sativum* and *Solanum lycopersicum*, the most intensively studied components of the organellar import systems are by far those of *Arabidopsis thaliana* (Stengel et al., 2009; Paul et al., 2013; Yan et al., 2014). *A. thaliana* is still the most appreciated model species for plant genomic research, most likely due to the availability of both the whole sequence of its small genome and unique genetic resources. Nevertheless, the difference between *A. thaliana* and other plant species is that forward and reverse functional genetics is highly implemented in *A. thaliana* due to the easy availability of an extensive collection of genetically modified loss- and gain-of-function lines. (Bouché and Bouchez, 2001). Because of this, *A. thaliana* has, by far, the best-annotated genome, which integrates annotations based on literature evidence together with curations from the scientific community (Berardini et al., 2015).

*Nicotiana benthamiana* is also emerging as an alternative tool for research in innate immunity and defense signaling during host-pathogen interaction. The main reason for this is its high susceptibility to many pathogens, such as viruses, bacteria, fungi, and many more. This particular feature makes it highly responsive to virus-based vectors and agrobacterium infiltration methods, which have been developed to express and purify foreign proteins, identificate protein-protein interactions and determine the subcellular localization of fluorescent protein-tagged proteins (Goodin et al., 2008; Bally et al., 2018). Concerning plant genomic research, sequences from two independent drafts assembly of the *N. benthamiana* genome, Sol Genomics Network and Nicotiana benthamiana Genome and Transcriptome Sequencing Consortium (benthgenome), began to be publicly available a decade ago but now are practically completed providing a considerable amount of information (Bally et al., 2018). Improved bioinformatics tools for the annotation of gene function are becoming increasingly available, but such information must be considered theoretical until further experimental evidence proves it. The arrival of the virus-induced gene silencing (VIGS) technique has dramatically accelerated the process, especially in the virus hypersusceptible *N. benthamiana*, by which plant molecular biologists can unravel the gene functions in other plants rather than *A. thaliana.* In addition, the ease with which *N. benthamiana* can be handled to generate stable transgenic lines and transiently express proteins has also facilitated rapid forward genetic screens. Despite all the above, the number of experimental validations of *N. benthamiana* gene functions found in the research literature is still minimal.

Here, we take advantage of the advances made in publicly available draft genomes and genome-based proteomes of *N. benthamiana* to identify the core components of the translocases of the outer membrane of chloroplasts and mitochondria. We also provide experimental evidence about their function based on their similarities with *A. thaliana* orthologs, phylogenetic analysis with its close relatives, identification of signatures in protein structures, subcellular localization, phenotypes of silenced plant, expression profiles at different tissues and stages, and characterization of physical interactions. Our findings will help to guide future studies about interactions between host preproteins and import machinery receptors to understand better the distinct organelle protein import pathways but, at the same time, to know how plant pathogens hijack host machinery for their own profit while taking advantage of the benefits of *N. benthamiana* as a research tool.

## Materials and methods

### Identification and gene amplification of the core components of the mitochondrion and chloroplast outer membrane translocases

The Arabidopsis protein and DNA sequences from the Arabidopsis Information Resource (TAIR) were used as query sequences to search for the putative core components of the mitochondrion and chloroplast outer membrane translocases using the genomes and predicted proteomes of *Nicotiana benthamiana* at the Sol Genomics Network (v1.0.1) (https://solgenomics.net) and *Nicotiana benthamiana* Genome and Transcriptome Sequencing Consortium (https://benthgenome.qut.edu.au). Functional domains were determined using BLASTp available through the National Center of Biotechnology Information, NCBI (https://www.ncbi.nlm.nih.gov). TPRs and transmembrane domains were predicted using TPRpred (https://toolkit.tuebingen.mpg.de/tools/tprpred) and the Dense Alignment Surface method (https://tmdas.bioinfo.se/), respectively. Molecular weights were estimated using Compute pI/Mw (https://web.expasy.org/compute_pi) (Karpenahalli et al., 2007). The coding region of NbToc90, NbToc120, NbToc159A, NbToc159B, NbToc34, NbTic22-III, NbToc75-III, NbTom20-1/-2, NbOm64 and NbTom40 were amplified by RT-PCR using SuperScript™ III One-Step RT-PCR Platinum™ Taq HiFi (Thermo Fisher Scientific, Carlsbad, CA, USA) and primers designed based on the ends of the gene sequences identified above (**Supplementary Table S1**). Total RNAs of *N. benthamiana*, isolated using EXTRAzol reagent following the producer’s protocol (BLIRT S.A., Gdańsk, Poland), were used as RT-PCR templates. The amplified fragments were cloned into appropriate vectors (see more detailed information about them and their usage in this section) and automatically sequenced at the IBMCP DNA Sequencing Service to assess matching with database entries. Pairwise amino acid identity comparison was made using SIAS (Sequence Identity And Similarity) (http://imed.med.ucm.es/Tools/sias.html).

### Phylogenetic analysis

The Toc and Tom receptor protein sequences from some *Nicotiana* sp. and *Solanum* sp. (family *Solanaceae*), *Arabidopsis thaliana*/*lyrata* (family *Brassicaceae*) and *Cucumis sativus* (family *Cucurbitaceae*) were retrieved from NCBI database (**Supplementary Table S2**). Amino acid sequences of *N. noctiflora* proteins were obtained from the *N. tabacum* phylome (entry 251, PhylomeDB database, http://phylomedb.org/). The coat protein of the melon necrotic spot virus (GenBank: DQ339157.1) was used as an outgroup. The multiple sequence alignment was performed in MEGA XI using ClustalW with default settings (Tamura et al., 2021). Evolutionary analysis was also conducted in MEGA XI by reconstructing the bootstrap consensus tree of sequences employing the minimum evolution method with 10000 bootstrap replicates. All branches corresponding to partitions reproduced in less than 40% of bootstrap replicates were collapsed.

### Molecular cloning

For subcellular localization studies, the coding regions of NbToc90/120/159A/159B, NbToc34, NbTic22-III, NbToc75-III, NbTom20-1/-2, NbOm64 and NbTom40, obtained as indicated above, were digested with the restriction enzymes shown in the **Supplementary Table S1** and fused in-frame to the 5′ or 3′ ends of the enhanced green fluorescent protein (GFP) by cloning them into a modified pBluescriptIIKS+ already harboring a duplicated cauliflower mosaic virus (CaMV) 35S promoter and the terminator of the potato protease inhibitor II (PoPit), pKS35S-PP. Depending on the presence of internal restriction sites, the expression cassettes were liberated by digestion either with SacI, HindIII, BsaI, or BsmbI and cloned into pMOG800 (Knoester et al., 1998). We used both bimolecular fluorescence complementation (BiFC) in plants and yeast two-hybrid (Y2H) assays for interaction studies. For BiFC, we proceed similarly to before. Either an amino-terminal fragment of the green fluorescent protein (GFP) (positions 1-155, Nt-[GFP]) or a carboxyl-terminal GFP fragment (positions 156-238, Ct-[GFP]) was either fused to the amino or carboxyl terminus of NbToc90/120/159A/159B and NbToc34 coding regions. Next, recombinant cDNAs were inserted into pKS35S-PP and further, the expression cassettes were transferred to pMOG800. For Y2H, NbToc90/120/159A/159B and NbToc34 coding regions were cloned into pGBKT7 (binding domain). The coding region of NbToc34 was also cloned in pGADT7 (activation domain) (Takara Bio USA, Inc., Mountain View, CA, USA). Oligonucleotides and restriction enzymes are listed in **Supplementary Table S1**.

### Agrobacterium tumefaciens-mediated transient expression and bimolecular fluorescence complementation assays

For transient expression assays in *N. benthamiana*, binary vectors obtained before were introduced into *Agrobacterium tumefaciens* strain C58C1 by electroporation. Transformed bacteria were grown overnight in liquid Luria-Bertani (LB) medium supplemented with kanamycin and rifampicin antibiotics. Cultures were pelleted and resuspended up to the required final OD600 value (0.2) with 10 mM MgCl_2_, 10 mM MES pH 5.6 and 150 µM acetosyringone. These suspensions were introduced in two-week-old leaves of *N. benthamiana* by gentle pressure infiltration into the abaxial side. For colocalization and BiFC experiments, which need the simultaneous expression of two different proteins, cultures were adjusted to an OD600 value of 0.5 and mixed in equal proportions before infiltration. Plants were grown under long-day photoperiods (16 h light at 25 °C and 8 h dark at 22 °C).

### Laser scanning confocal microscopy, subcellular fluorescent markers and image analysis

The fluorescent-tagged protein subcellular localization and BiFC were visualized with an inverted Zeiss LSM 780 laser scanning confocal microscope (Carl Zeiss, Oberkochen, Germany) two days after the agroinfiltration. eGFP and cherry fluorescent protein (ChFP) excitation were done using 488 and 561 nm wavelength lasers, respectively. The windows for emission detection were set between 492–532 nm for GFP and 590–630 nm for ChFP. The chlorophyll excitation wavelength was 488 nm, and emission was collected above 700 nm. Transit peptide of yeast cytochrome oxidase subunit IV (coxP, matrix) fused to a cherry fluorescent protein (ChFP) N-terminus (coxP-ChFP) was used as a mitochondrial matrix marker. In addition, glyrsP-ChFP consisting of the transit peptide of the *A. thaliana* glycyl-tRNA synthetase fused to the ChFP was used as a chloroplast and mitochondrion dual marker (Navarro et al., 2021). Image processing and analysis, including overlays and Z-stack projections, were performed using FIJI software (Schindelin et al., 2012).

### Yeast two-hybrid assay

Y2H assays were performed using the GAL4-based MATCHMAKER Two-Hybrid System following the manufacturer’s protocol (Fields and Song, 1989) (Takara Bio USA, Inc., Mountain View, CA, USA). Full-length ORFs of NbToc90, NbToc120, NbToc159A and NbToc159B and NbToc34 were used as prey and cloned into pGADT7 (pGADNbToc90, pGADNb120, pGADNb159A, pGADNb159B and pGADNbToc34). NbToc34 ORF was also cloned into pGBDKT7 to be used as a bait vector (pGBNbToc34) and transformed into AH109 yeast cells. Next, all pGAD constructs were transformed into AH109 cells already containing pGBNbToc34. Putative interactions were analyzed by culturing co-transformants on SD+DO-A-H-L-W+X-α Gal. To confirm the specificity of the interactions, all pGAD constructs were also transformed in yeast cells harboring pGBKT7 or pBD-p53 (tumor protein p53).

### Viral-induced gene silencing in *A. thaliana*

For VIGS, pTRV1 and pTRV2 Gateway vectors were used (Liu et al., 2002). A target region of about 300 bp from NbToc90/120/159A/159B, NbToc34, NbTic22-III, NbToc75-III, NbTom20-1/-2, NbOm64 and NbTom40 genes was selected using SGN VIGS Tool (Fernandez-Pozo et al., 2015). Fragments were PCR amplified with gene-specific oligonucleotides (**Supplementary Table S1**) and then recombined with pDONR207. The resultant pENTRY vectors were recombined with pTRV2 according to the manufacturer’s instructions (Invitrogen Life Tech, Carsland, CA, USA). A pTRV2 carrying the complete mGFP5 gene, pTRV2[GFP] (Navarro et al., 2020), was used as control. pTRV1 and all pTRV2 derivates were introduced into *A. tumefaciens* by electroporation. Agroinfiltrations for VIGS were done using the leaf infiltration method in *N. benthamiana* as described above, but, on this occasion, bacterial cultures were resuspended to a final OD600 of 1. Two-week-old *N. benthamiana* seedlings were infiltrated with bacterial cultures carrying pTRV1 and each pTRV2 derivated mixed in a 1:1 volume ratio. Gene silencing was monitored using real-time quantitative reverse transcription PCR over ten days after infiltration. Plants were grown under long-day photoperiods (16 h light at 25 °C and 8 h dark at 22 °C).

### Real-time quantitative reverse transcription PCR

Total RNA from at least three silenced plants of each gene was obtained by extraction with RIBOzol Reagent. Remnant genomic DNA was removed by DNase I treatment. First-strand cDNA was synthesized from 0.5 µg of total RNA using RevertAid H Minus Reverse Transcriptase and oligo(dT) (Thermo Fisher Scientific, Carlsbad, CA, USA). Real-time qPCR was carried out using QuantStudio 3 Real-Time PCR machine (Applied Biosystems, Waltham, MA, USA) and PyroTaq EvaGreen qPCR Supermix (Solis BioDyne, Tartu, Estonia), specific oligonucleotides, and recommended qPCR cycles as follows: initial denaturation for 12 min at 95°C, followed by 50 cycles of 15 s at 95°C and 60 s at 60°C. Specific oligonucleotides were designed using Primer3web version 4.1.0 (https://bioinfo.ut.ee/primer3). Oligonucleotide efficiencies were tested by qRT-PCR using tenfold serial dilutions of the corresponding cDNA. Each biological replicate was run in triplicate. Elongation factor 1-α (EF1α, TC19582), F-BOX family protein (F-BOX, Niben.v0.3.Ctg24993647) and protein phosphatase 2A (PP2A, TC21939) genes were used as endogenous controls (Liu et al., 2012).

## Results

### Identification of the receptors and the main channel component of Toc and Tom from *N. benthamiana.*

**Figure 1:**
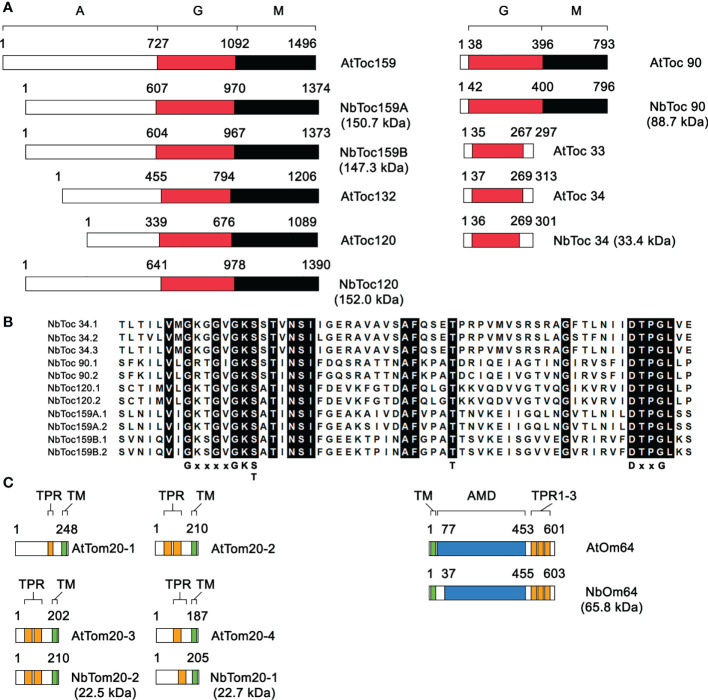
Structural comparison of the core components of the Toc and Tom complexes in *N. benthamiana* and **(*A*)** *thaliana*. **(A)** Schematic alignment of the domain organization of Toc34 and Toc159 family receptors in *N. benthamiana* and **(*A*)** *thaliana*. The positions of the acidic domains (A, white boxes), the GTPase domains (G, red boxes) and the membrane anchor domain (M, black boxes) are shown. The sizes of the *N. benthamiana* orthologs in kDa are indicated to the right of each representation. The numbers above each drawing correspond to the amino acid positions at the domain boundaries. **(B)** Amino acid alignment of the region of the G domains of the *N. benthamiana* Toc receptors holding the P-loop (G1) motif (GxxxxGKS/T), the threonine in switch-I (G2) and the residues DxxG of switch-II (G3). **(C)** Schematic alignment of the domain organization of isoforms of Tom20 and Om64 receptors in *N. benthamiana* and **(*A*)** *thaliana*. The positions of the transmembrane domains (TM, green boxes), the tetratricopeptide repeats (TPR, orange boxes) and the amidase domain (AMD, blue boxes) are shown. The sizes of the *N. benthamiana* orthologs in kDa are indicated to the right of each representation. The numbers above each drawing correspond to the amino acid positions at the domain boundaries.

To identify the putative receptors and the main channel component of Toc and Tom complexes in *N. benthamiana*, a BLASTp search was conducted using *A. thaliana* amino acid (aa) sequences (**Supplementary Table S2**) and the *N. benthamiana* genome v1.0.1 (available at Sol Genomics Network, https://solgenomics.net). The aa sequences of *N. benthamiana* showing the highest identity were tentatively designated as NbToc34, NbToc90, NbToc120, NbToc159A, NbToc159B, NbTic22-III, NbTic22-IV, NbToc75-III, NbTom20-1, NbTom20-2, NbOm64 and NbTom40 (**Figure 1** and **Table 1**). At least two *N. benthamiana* aa sequences, denoted by a number (e.g., NbToc34.1 and NbToc34.2), were identified for each *A. thaliana* query protein. The corresponding gene structures were visualized using the Genome Browser Tool (https://solgenomics.net) and the Niben v1.0.1 genome. The size and number of exons of each pair of genes were similar or identical. Still, they differed in intron/exon arrangement as well as in their chromosome location (NbLab330 database) and, occasionally, in their transcriptional orientation (**Supplementary Figure 1** and **Supplementary Table S3**). All of them were supported by mRNA-seq evidence in the Niben v1.0.1 gene model and they also matched with NibSet-1 and NbDE protein datasets with a minimum sequence identity of 94.3% (**Supplementary Table S3**) (Kourelis et al., 2019; Schiavinato et al., 2019). These findings indicated that each pair of sequences are not caused by polymorphisms between different sequenced samples and assembly errors but corresponded to different gene copies.

**Table 1:** Amino acid sequence identity among Tom and Toc receptors of *Nicotiana benthamiana* and *Arabidopsis thaliana*.

	AtTom20-1	AtTom20-2	AtTom20-3	AtTom20-4	AtOm64	
**NbTom20-1**	42.92%	57.07%	58.91%	59.35%		
**NbTom20-2.1** **NbTom20-2.2**	41.08% 42.57%	56.93% 56.93%	60.39% 58.41%	58.28% 57.75%		
**NbOm64** **NbOm64-like**					64.45% 49.39%	
	**AtToc90**	**AtToc120**	**AtToc132**	**AtToc159**	**AtToc34**	**AtToc33**
**NbToc90.1** **NbToc90.2**	51.95% 51.96%	38.31% 40.02%	38.06% 39.89%	34.54% 37.13%	27.15% 28.43%	25.92% 26.93%
**NbToc120.1** **NbToc120.2**	38.83% 38.83%	60.51% 60.33%	56.68% 56.59%	32.08% 32.23%	28.75% 28.11%	26.93% 26.93%
**NbToc159A.1** **NbToc159A.2**	33.03% 32.03%	31.31% 31.22%	30.53% 30.29%	41.55% 39.62%	29.07% 28.75%	27.94% 27.60%
**NbToc159B.1** **NbToc159B.2**	34.42% 34.17%	36.17% 35.72%	34.10% 34.27%	52.65% 49.63%	26.19% 26.19%	26.59% 26.59%
**NbToc34.1** **NbToc34.2**	26.57% 25.41%	29.90% 29.37%	30.56% 30.03%	30.23% 29.04%	67.44% 64.35%	61.95% 60.26%
	**AtTic22-III**	**AtTic22-IV**		**AtTom40**	**AtToc75-III**	
**NbTic22-III.1** **NbTic22-III.2**	61.85% 58.87%	29.62% 28.62%	**NbTom40.1** **NbTom40.2** **NbTom40.3** **NbTom40.4**	73.07% 72.75% 68.80% 69.11%		
**NbTic22-IV.1** **NbTic22-IV.2**	27.79% 26.55%	59.57% 53.28%	**NbToc75-III.1** **NbToc75-III.2**		78.28% 77.01%	
	**A domain**		**G domain**		**M domain**	
**NbToc90 vs** **AtToc90**			54.31%		49.49%	
**NbToc120 vs** **AtToc120**	11.2%		86.35%		71,35%	
**NbToc120 vs** **AtToc132**	11.64%		87.24%		71.84%	
**Toc159A vs** **AtToc159**	16.47%		55.09%		65.59%	
**Toc159B** **Vs AtToc159**	21.02%		69.14%		73.64%	

Underlined values correspond to the highest value of aa sequence identitiy.

Besides, *N. benthamiana* contains a complex allotetraploid genome formed by interspecific hybridization of two diploid progenitors. The maternal ancestor was probably a species in section *Noctiflorae*, which introgressed some DNA from a species in section *Petunioides* (*N. attenuate*), while the paternal parent belonged to section *Sylvestres* (Schiavinato et al., 2020). This means that at least two homeologs per gene could be found (Bally et al., 2018). Schiavinato et al. used phylogenetic distances to assign a parental origin to the *N. benthamiana* genes (NibSet-1 gene models, http://bioinformatics.boku.ac.at/NicBenth/Download/) (Schiavinato et al., 2019) and scaffolds (Sol Genomics Network). Based on their data, we analyzed the parental origin of all the identified proteins in this work. Except for NbTic22-IV aa sequences, which had a paternal origin, a maternal source was assigned to the rest, indicating that each pair of sequences are not homeologs (**Supplementary Table S3**). This is possible because new hybridizations during the pseudodiploidization process led to losses in some genomic regions and even whole chromosomes (Bally et al., 2018). Nevertheless, phylogenetic relationships to other Toc and Tom receptors from related and distant species suggested a close relationship between most of the analyzed *N. benthamiana* sequences and their parental species (**Supplementary Figures 2–5**).

In *A. thaliana*, the Toc receptors either belong to Toc34 or Toc159 families consisting of two or four different genes coding for AtToc33/34 and AtToc90/120/132/159, respectively (**Figure 1A**). Four aa sequences of a putative *N. benthamiana* ortholog of AtToc34 were found (NbToc34.1-4). NbToc34.1 and NbToc34.3, as well as NbToc34.2 and NbToc34.4, were near-identical aa sequences (**Supplementary Table S3**). However, structural similarities such as the size and number of exons as well as transcriptional orientation were higher between the corresponding genes of NbToc34.1 and NbToc34.2 and between NbToc34.3 and NbToc34.4, but some indels were observed only in the last pair. It is noteworthy that *NbTOC34.1-3* had a maternal origin while *NbTOC34.4* was declared “orphan”; thus, they most likely arose from recent gene duplication events. NbToc34.1 and NbToc34.2 shared 67.44% and 64.35% overall aa sequence identity, respectively, with AtToc34 but 61.95% and 60.26% with AtToc33 (**Table 1**). Thus, we considered both as NbToc34 orthologs. The phylogenetic tree generated in this study, which includes some *Nicotiana* sp. and *Solanum* sp. (family *Solanaceae*), *A. thaliana*/*lyrata* (family *Brassicaceae*) and *Cucumis sativus* (family *Cucurbitaceae*) sequences retrieved from NCBI database, showed that Toc34 aa sequences of members of the family *Solanaceae* grouped into two clades (80% bootstrap support), and NbToc34.1 and NbToc34.2 fell into each one of them (**Supplementary Figure 2**).

Four pairs of aa sequences were found concerning putative orthologs belonging to the Toc159 family (**Supplementary Table S3**). One of them showed an estimated molecular weight of about 88.7 kDa (**Figure 1A**) and shared the highest overall aa sequence identity with AtToc90 (NbToc90.1, 51.95% and NbToc90.2, 51.96%, **Table 1**). Both sequences fell into a clade (100% bootstrap support) exclusively composed of Toc90 samples from species of the genus *Nicotiana* (**Supplementary Figure S2**). The members of the *A. thaliana* Toc159 family were numbered according to their molecular weight; thus, based on their predicted molecular masses of about 150 kDa (**Figure 1A**), the remaining three pairs of *N. benthamiana* aa sequences could correspond to AtToc159 orthologs. However, one of them shared the highest identity in the whole sequence with AtToc120 (60.51% and 60.33%) and AtToc132 (56.68% and 56.59%). The aa sequence identity was even higher for the G (86,35% and 87.24%) and D (71.35% and 71,84%) domains (**Table 1**). We also show that they fell into a clade (100% bootstrap support) exclusively composed of aa sequences from the genus *Nicotiana* that were annotated as Toc120 in the NCBI database (**Supplementary Figure S2**) and related with AtToc120/132. Following the trend of what has been done in other orthologs of similar and higher sizes from *Nicotiana* and *Solanum* species, we named them NbToc120.1 and NbToc120.2 (**Supplementary Table S3**). The other two pairs of deduced aa sequences shared an identity ranging from 39.62% to 52.65%, approximately, with AtToc159 (**Table 1**). As they are also clustered in two divergent clades, they are both likely to be different Toc159 isoforms to which we arbitrarily assigned the names NbToc159A and NbToc159B. A third sequence, NbToc159A.3, sharing a high identity with NbToc159A.1 and NbToc159A.2 was also identified but lacking near 300 aa in its amino end. We also constructed a phylogenetic tree with orthologs of AtTicIII or AtTicIV from some species belonging to the genera *Solanum* and *Nicotiana* found in the NCBI search. Sequences were equally distributed into two groups (99% bootstrap support), each including either AtTicIII or AtTicIV (**Supplementary Figure S3**).

Despite the difference in their molecular sizes, all Toc receptors share a central conserved GTPase domain (G-domain) and members of the Toc159 family also share a conserved C-terminal membrane anchor domain (M-domain) and a variable N-terminal acidic domain (A-domain), which in *A. thaliana* is also variable in length (AtToc120: 339 aa; AtToc132: 455 aa, AtToc159: 727 aa). In contrast, the A domain of NbToc120 (641 aa), NbToc159A (607 aa), and NbToc159B (604 aa) showed a similar length but still a low degree of sequence identity (8.23%-14.07%) (**Figure 1A**). Analysis of *N. benthamiana* aa sequences of Toc receptors using the NCBI Conserved Domain Database (http://www.ncbi.nlm.nih.gov/Structure/cdd/wrpsb.cgi) confirmed the presence of the G domain (cl38936: P-loop_NTPase) with the five canonical guanine nucleotide-binding motifs (G1-5). The P-loop (G1) motif (GxxxxGKS/T), the threonine in switch-I (G2) and the residues DxxG of switch-II (G3) were conserved (**Figure 1B**). In addition to the G domain, the M domain (pfam11886, TOC159_MAD) was also identified in NbToc90, NbToc120 and NbToc159A/B, confirming that all of them have the same characteristic tripartite domain organization.

Regarding mitochondria, Tom20 is the principal receptor of the Tom complex, and in *A. thaliana*, there are up to four isoforms of Tom20 (AtTom20-1 to AtTom20-4). AtTom20 belongs to the tetratricopeptide repeat (TPR) superfamily and, contrary to what happens in animals and fungi, it is anchored to the mitochondrial outer membrane through a C-terminal transmembrane domain (Ghifari et al., 2018) (**Figure 1C**). Only two ortholog proteins, which we have designed as NbTom20-1 (22.7 kDa) and NbTom20-2 (22.5 kDa), were identified by searching in both Sol Genomics Network and the Nicotiana benthamiana Genome and Transcriptome Sequencing Consortium (https://benthgenome.qut.edu.au). *TOM20-1.1* and *TOM20-2.1/2* have six exons of nearly identical sizes, but the first two were absent in *NbTOM20-1.2.* Interestingly, *TOM20-2.1* and *TOM20-2.2* showed different transcriptional orientations. Our phylogenetic analyses also revealed that almost all *Nicotiana* sp. sequences were separated into two clusters (83% bootstrap support), each of which included one of the *N. benthamiana* variants (**Supplementary Figure S4**). Amino acid sequence comparison of NbTom20-1 and NbTom20-2 with *A. thaliana* isoforms revealed that the highest identity was with AtTom20-4 (59.35%) and AtTom20-3 (60.39%-58.41%), respectively (**Table 1**). According to motif prediction by TPRpred, two TPR motifs were predicted in both AtTom20-3 and NbTom20-2 in a similar position (41-74 and 86-119 vs 41-74 and 79-112, respectively), but only one in both AtTom20-4 (81-116) and NbTom20-1 (79-114). A hydrophobic region was also predicted at the Ct of both NbTom20-1 (positions 178-192) and NbTom20-2 (positions 174-187) using the Dense Alignment Surface method (**Figure 1B**).

The 64-kDa outer envelope protein, Om64, is not present in yeast or mammals, but it seems to play a role as an import receptor in some vascular plants (Carrie et al., 2010). Here, we found three putative *N. benthamiana* orthologs of 58.98, 62.43 and 65.80 kDa that were annotated just as Glutamyl-tRNA(Gln) amidotransferase subunit A and showed 64.45%, 47.10% and 49.91% amino acid identity with AtOm64. The smallest two differed in the insertion of 45 aa but were highly similar in shared sequence (95.87%). However, they differed quite from the largest ones (49.82% and 50.53%). In *A. thaliana*, the aa sequence of AtOm64 is about 51% identical to the chloroplast localized AtToc64-III. Thus, the smallest two may correspond to orthologs of AtToc64-III, with which they share a higher amino acid sequence identity (63.10% and 66.50%) (**Table 1** and **Supplementary Table S3**). In this sense, both aa sequences clustered with AtToc64-III while the third sequence, which was then named NbOm64, grouped with AtOm64 and other Om64 aa sequences from the genera *Solanum* and *Nicotiana* (97% bootstrap support) (**Supplementary Figure S4**). In *A. thaliana*, AtOm64 has an N-terminal transmembrane anchor region followed by a globular cytosolic region that shows sequence similarity to an amidase and contains three TPR at the C-terminus (Carrie et al., 2010) (**Figure 1B**). Analysis of NbOm64 using the NCBI Conserved Domain Database confirmed the presence of the amidase superfamily domain (pfam01425) and the three TPR (positions 486-518, 521-552 and 554-580). A putative transmembrane domain was also predicted at its Nt (positions 15-29) using the Dense Alignment Surface method (**Figure 1C**).

Finally, Toc75 and Tom40 are the central channels for protein translocation across the chloroplast and mitochondria outer membranes, respectively. Both are transmembrane β-barrel proteins. In addition, Toc75 also contains three POTRA domains, followed by the C-terminal membrane-spanning β-barrel (Rapaport and Neupert, 1999; Panigrahi et al., 2015). In *A. thaliana*, four Toc75-related genes have been identified, AtToc75-I, AtToc75-III, AtToc75-IV and AtToc75-V -(renamed AtOEP80), but only AtToc-III is proposed to form the protein conducting channel in Toc complexes (Baldwin et al., 2005). Two sequences corresponding, as mentioned above, to a unique putative ortholog of AtToc75-III in *N. benthamiana* (**Supplementary Figure S1**) were found to share 92.2% identity between them and 78.28% and 77.01% aa sequence identity with AtToc75-III. As expected, both clustered into the group of Nicotiana sequences (99% bootstrap support) (**Supplementary Figure S5**). As putative *N. benthamiana* orthologs of AtTom40, we found four similar aa sequences near-identical two by two in sequence and exon structure of its corresponding genes, NbTom40.1/2 and NbTom40.3/4 (**Figure S1** and **Supplementary Table S3**). Although a maternal origin was assigned for all of them, phylogenetic analysis showed that NbTom40.1/2 and NbTom40.3/4 were grouped with *N. noctiflora* and N. sylvestris, respectively (**Supplementary Figure S5**).

### *Nicotiana benthamiana* and *A. thaliana* orthologs show identical subcellular localization after transient expression

**Figure 2:**
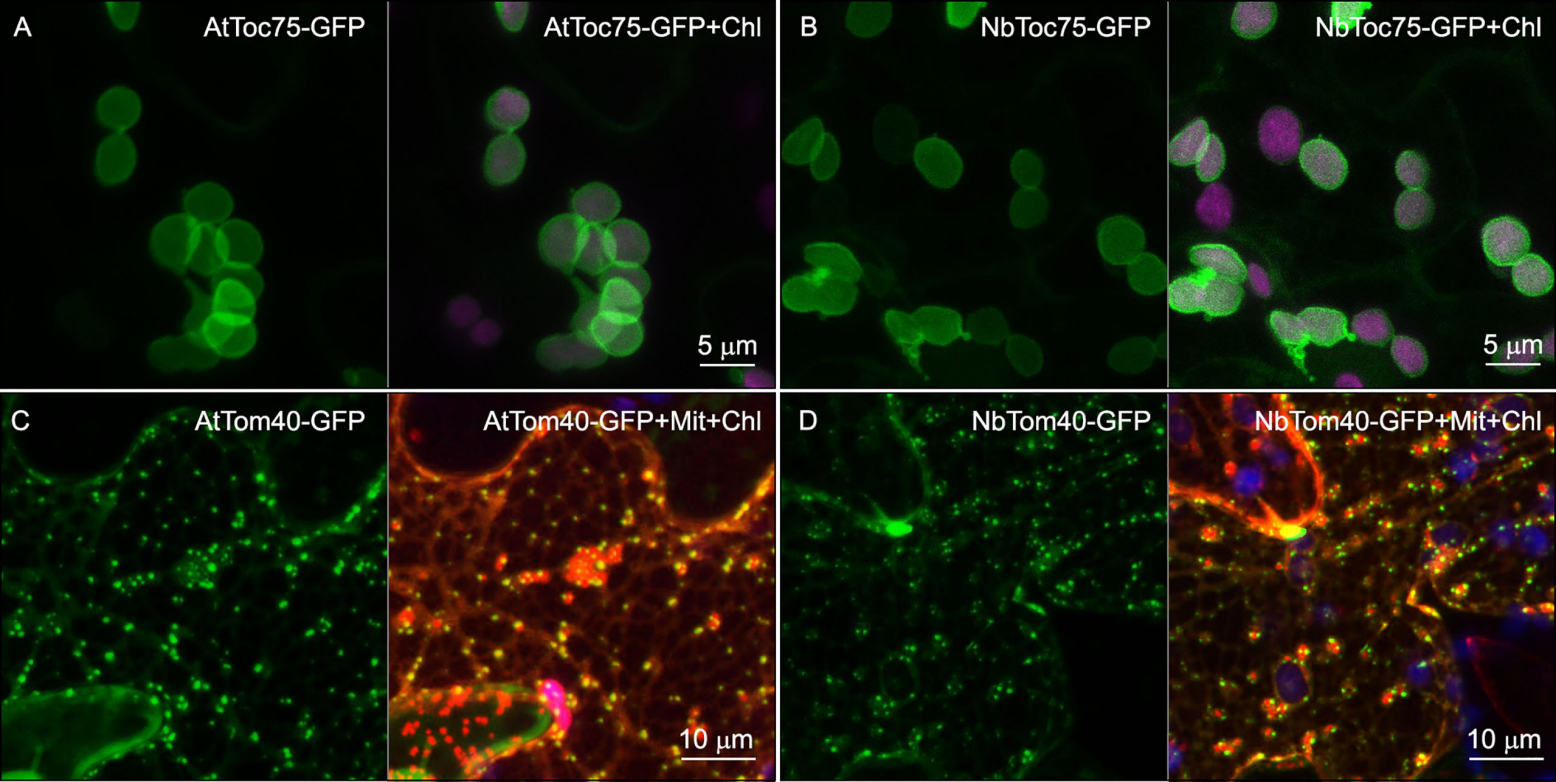
Subcellular localization of Toc (Toc75-III) and Tom (Tom40) channels of *N. benthamiana* and *A. thaliana*. GFP fusion proteins (green channel), indicated in the upper part of each panel, were expressed in epidermal cells of *N. benthamiana* by transient expression mediated by agrobacterium. All images correspond to Z-stack projections taken two days after infiltration. Chlorophyll fluorescence is shown in magenta (Chl). To better visualize the rim of fluorescence surrounding the chloroplast in **(A)** and **(B)**, the green channel alone (left panel) and merged with the magenta channel (right panel) are shown. The red channel in **(C)** and **(D)** corresponds to the mitochondrial matrix marker, coxP-ChFP (Mit).

**Figure 3:**
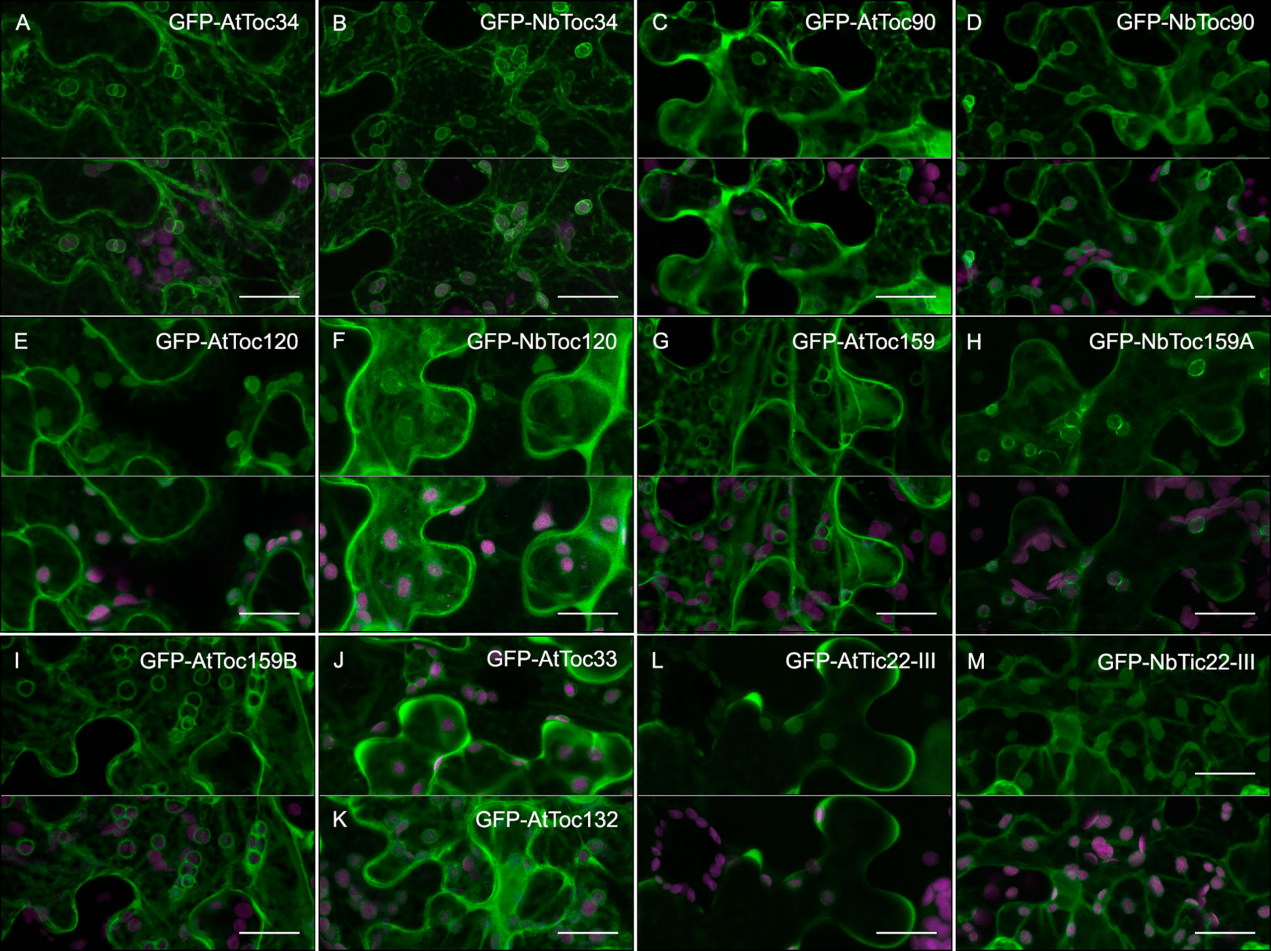
Subcellular localization of Toc receptors of *N. benthamiana* and *A. thaliana* **(A–M)**. GFP fusion proteins (green channel), indicated in the upper part of each panel, were expressed in epidermal cells of *N. benthamiana* by transient expression mediated by agrobacterium. All images correspond to Z-stack projections taken two days after infiltration. Chlorophyll fluorescence is shown in magenta. To better visualize the rim of fluorescence surrounding the chloroplast, the green channel alone (upper part of each panel) and merged with the magenta channel (bottom part of each panel) are shown. Scale bars correspond to 20 µm.

**Figure 4:**
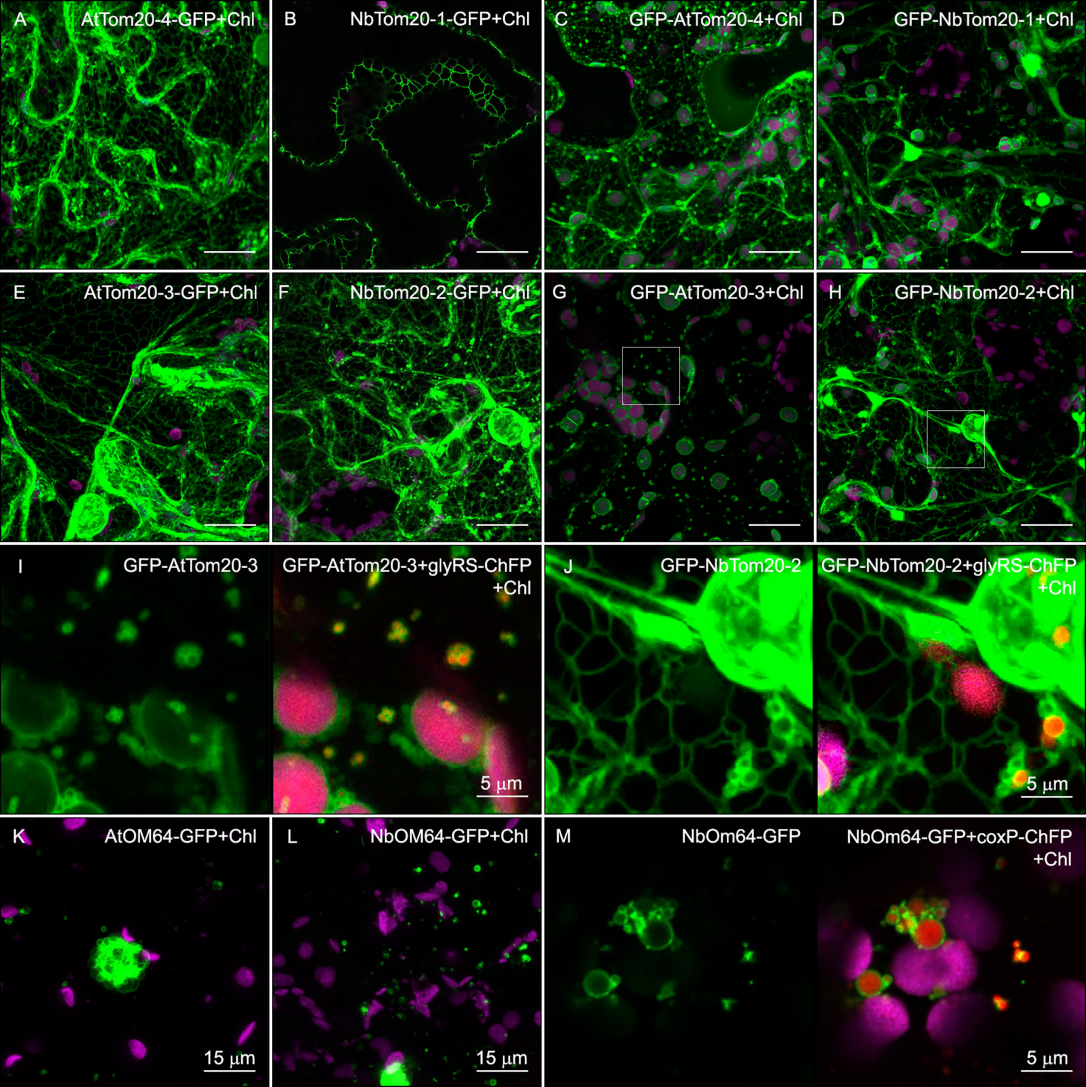
Subcellular localization of Tom receptors of *N. benthamiana* and *A. thaliana*. GFP fusion proteins (green channel), indicated in the upper part of each panel, were expressed in epidermal cells of *N. benthamiana* by transient expression mediated by agrobacterium **(A–M)**. All images correspond to Z-stack projections taken two days after infiltration. The red channel either corresponds to the mitochondrion and chloroplast dual marker glyRS-ChFP or the matrix marker, coxP-ChFP, as indicated. Chlorophyll fluorescence is indicated (Chl) and shown in magenta. **(I, J)** panels correspond to a magnification of the area delimited by the box in panels **(G, H)**, respectively. Scale bars correspond to 20 µm unless indicated.

Subcellular localization largely influences protein function by controlling interactor accessibility. Thus, to complete our bioinformatics analysis, we evaluated the subcellular localization of the *N. benthamiana* Toc and Tom receptors and channels identified above, making a comparison with *A. thaliana* ortholog proteins in plant cells. To do that, cDNA of the *N. benthamiana* proteins denoted by number one in **Supplementary Table S3**, in addition to their *A. thaliana* orthologs, were RT-PCR amplified and cloned into the appropriate vectors. Except for Toc75-III and Tom40, the green fluorescent protein (GFP) was fused to their C- or N-terminus, and next, they were transiently expressed in *N. benthamiana* leaves. The fluorescence was visualized under laser scanning confocal microscopy (LSCM) at 48 hpi, but C-terminally GFP-tagged Toc159 family members produced a very weak or no fluorescent signal. It has been reported that adding a bulky tag may mask the sorting information, which is localized at the Ct of the Toc receptors from both Toc34 and Toc159 families, resulting in the proteasome-mediated degradation of the mistargeted fusion proteins (Lung and Chuong, 2012). Nevertheless, fluorescence was visualized properly in the corresponding fusions harboring an N-terminal GFP (**Figures 2**–**4**).

Regarding Toc75‐III and Tom40, Toc75-III has a bipartite Nt targeting signal that is cleaved by the stromal and the intermembrane space localized type I signal peptidase (Tranel and Keegstra, 1996), and, although the Tom40 targeting signal has not been still resolved, it was shown that the fusion of GFP at the N terminus of human Tom40 abolished its mitochondrial targeting (Humphries et al., 2005). Therefore, we only analyzed the C-terminally fused versions (**Figures 2A–D**). AtToc75-III and NbToc75-III gave a circular, rim-like fluorescence pattern surrounding the chlorophyll fluorescence (pseudocolored in magenta), probably located on the chloroplast surface, that is consistent with the localization of AtToc75-III in the chloroplast outer membrane (**Figures 2A, B**). AtTom40-GFP and NbTom40-GFP were observed in small punctate structures within *N. benthamiana* epidermal cells. Visualization of mitochondria in these cells with the mitochondrial matrix marker coxP-ChFP (in red) revealed that many punctate structures were associated with these organelles (**Figures 2C, D**).

In addition to chloroplast outer envelope localization, the *A. thaliana* and *N. benthamiana* Toc receptors of both Toc34 and Toc159 families fused to the GFP C-terminus were also abundant in the cytoplasm, indicating that they partition between soluble and membrane fractions (**Figures 3A-K**). A similar pattern was observed with Nt and Ct GFP fusions of AtTic22-III and NbTic22-III, but more diffuse fluorescence throughout chloroplast was observed instead of forming the rim-like pattern (**Figures 3L, M** and **Supplementary Figure S6**). AtToc33-GFP, AtToc34-GFP and NbToc34-GFP mainly produced diffuse fluorescent signals in the cytoplasm and, only occasionally, showed the rim-like distribution around chloroplasts (**Supplementary Figure S6**).

Expression of *A. thaliana* and *N. benthamiana* isoforms of Tom20 harboring a C-terminal GFP tag resulted in endoplasmic reticulum (ER) mistargeting (**Figures 4A, B, E, F** and **Supplementary Figure S6**). GFP N-terminally tagged versions were also located in the ER. Still, they mainly produced rims surrounding the chloroplasts (chlorophyll fluorescence pseudocolored in magenta) and mitochondria (chloroplast and mitochondrion dual marker, glyRS-ChFP in red) (**Figures 4C, D, G–J** and **Supplementary Figure S6**). Overexpression of both AtOm64-GFP and NbOm64-GFP produced fluorescent vesicles of different sizes evenly distributed over the cytoplasm and occasionally aggregated (**Figures 4K, L**). Upon coexpression with coxP-ChFP, red fluorescence was observed inside the vesicles indicating that they correspond to mitochondria that have suffered some swallowing alteration, most likely due to membrane protein overexpression (**Figure 4M**). In contrast to AtOm64-GFP and NbOm64-GFP, which seem to be targeted appropriately to the outer mitochondrial membrane, GFP-AtOm64 and GFP-NbOm64 produced a cytoplasmic fluorescent pattern (**Supplementary Figure S6**). GFP tagging to the AtOm64 and NbOm64 N-termini probably disrupted their mitochondrial anchoring that is mediated by their Nt transmembrane domains.

### Developmental phenotypes of *N. benthamiana* plants silenced for genes encoding Tom/Toc receptors and channels

**Figure 5:**
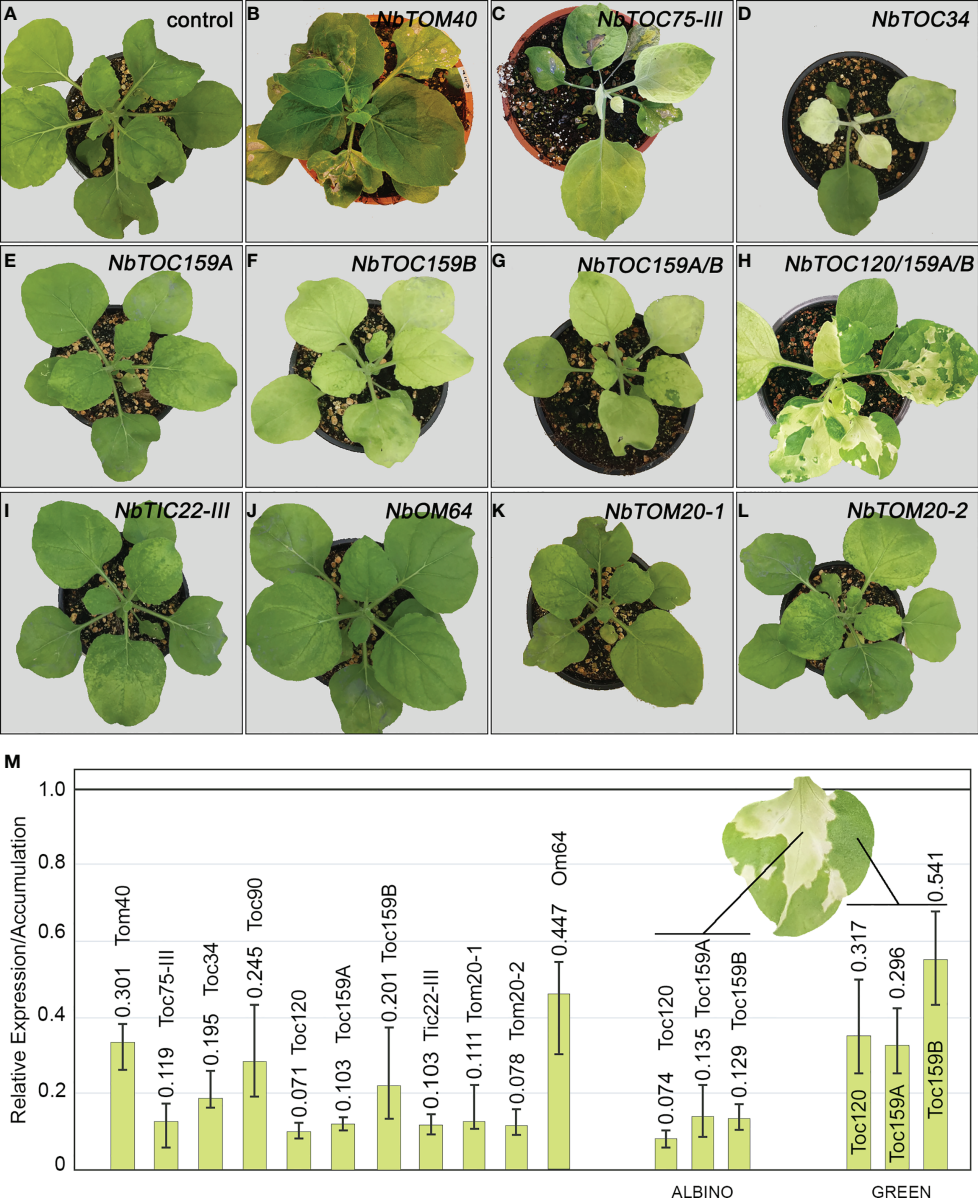
Analysis of the phenotype of the *N. benthamiana* plants silenced for each core component of the Toc and Tom complexes using viral-induced gene silencing mediated by TRV. **(A–L)** Phenotype of the *N. benthamiana* plants silenced for each indicated gen. Images were taken three weeks after infiltration with pTRV1 and each variant of the pTRV2 construct. Control corresponds to a plant infiltrated with pTRV1 and pTRV2[GFP]. The phenotype of both *NbTOC90-*, and *NbTOC120-*silenced plants were as control. **(M)** Relative gene expression of *NbTOM40*, *NbTOC75-III*, *NbTOC34*, *NbTOC90*, *NbTOC120*, *NbTOC159A*, *NbTOC159B*, *NbTIC22-III*, *NbTOM20-1*, *NbTOM20-2* and *NbOM64* in the corresponding *N. benthamiana* silenced plants. The relative gene expression of NbTOC120, NbTOC159A and NbTOC159B in green and albino regions from variegated leaves is shown on the right. An RNA mix from three different plants was used in each case. Expression levels in the control plant were used as reference sample. The error bars indicate the RQ minimum and maximum.

To go deeper into the function of the individual Toc/Tom receptors and channels in *N. benthamiana*, we decided to silence them using a viral-induced gene silencing (VIGS) approach (Liu et al., 2002). We were able to generate VIGS constructs with a tobacco rattle virus binary system (pTRV1 plus pTRV2 vectors) that have the potential to target all gene copies. pTRV2 carrying the complete mGFP5 gene, pTRV2[GFP], was used as control. VIGS was performed with 2 weeks old *N. benthamiana* plants. Three weeks later, silenced plants were compared with control plants. *NbTOC90-*, *NbTOC120-*, *NbTOC159A-* (**Figure 5E**), *NbTIC22-III-* (**Figure 5I**), *NbOM64-* (**Figure 5J**), *NbTOM20.1-* (**Figure 5K**) and *NbTOM20-2-* (**Figure 5L**) silenced plants showed no visible phenotype except for the typical TRV symptoms, such as mildly curved leaves and a very slight but unevenly distributed chlorosis, mainly in young leaves (**Figure 5A**). In contrast, *NbTOM40*-, *NbTOC75-III*-, *NbTOC34*- and *NbTOC159B*-silenced plants showed different developmental phenotypes depending on the silenced gene. *NbTOM40*-silenced plants showed leaf and petiole necrosis that produced some constriction sites, finally causing the leaf to wither and die. Plant growth was also arrested, most likely due to this phenotype (**Figure 5B**). The VIGS lines for *NbTOC75-III* (**Figure 5C**) and *NbTOC34* (**Figure 5D**) showed similar strong albino phenotypes with occasional necrosis around the veins, dwarfism of newly emerging leaves and severe arrested growth. In contrast, the *NbTOC159B*-silenced plants only had a slightly pale phenotype. Silencing of both *NbTOC159A* and *NbTOC159B* did not accentuate the phenotype of *NbTOC159B-*silenced plants (**Figure 5E**–**G**). Still, the silencing of both *NbTOC159A* and *NbTOC159B* plus *NbTOC120* led to a variegated phenotype with green and albino leaf regions and occasional distortion (**Figure 5H**).

To check the expression levels of the targeted genes, quantitative RT-PCR was performed using gene-specific primers. Infiltrated pTRV2[GFP] plus pTRV1 plants were used as a positive control. The mRNA expression levels of all genes were reduced in silenced plants compared to the levels in control plants three weeks after TRV infection. Although the silencing levels varied, the silencing efficiency ranged from 70% (NbTom40) to 93% (NbToc120), except for Om64, for which we reached up to 55% at best. Albino and green leaf regions in *NbTOC120/159A/159B* silenced plants were associated with high and moderate levels of gene silencing, respectively (**Figure 5M**).

### Expression profiles of the Toc and Tom receptors from *N. benthamiana*

**Figure 6:**
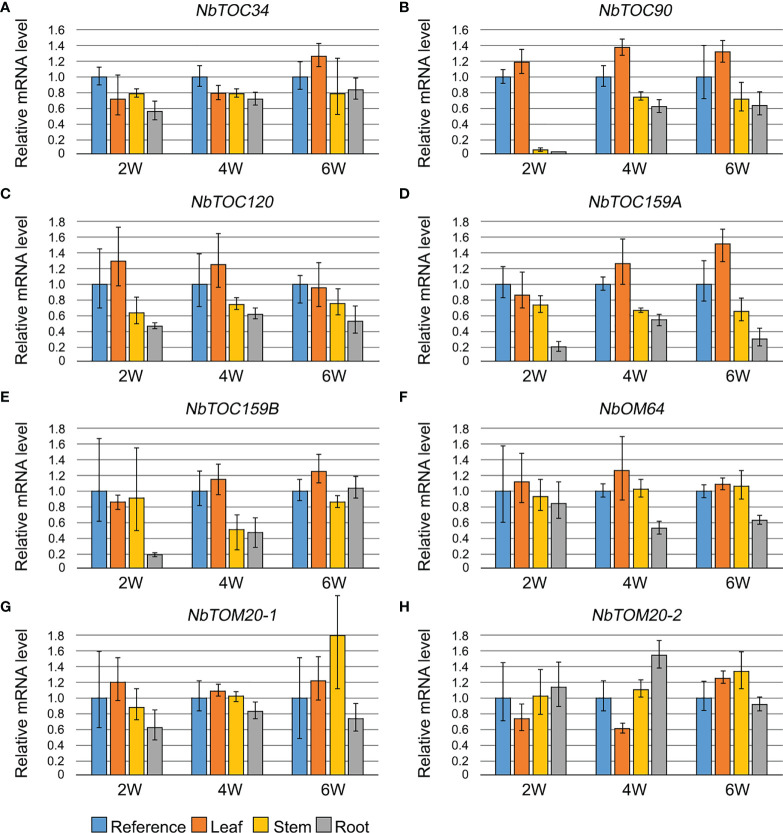
Relative gene expression of *NbTOC34***(A)**, *NbTOC90***(B)**, *NbTOC120***(C)**, *NbTOC159A***(D)**, *NbTOC159B***(E)**, *NbOM64***(F)***, NbTOM20-1* **(G)** and *NbTOM20-2* **(H)** genes in leaves (orange), stems (yellow) and roots (cool grey) analyzed by RT-qPCR at two (2W), four (4W) and six (6W) weeks after germination. The mean value from three different plants is shown. Total RNAs extracted from a mix of leaves, stems and roots was used as reference sample (blue). Error bars correspond to ± SD.

The knowledge of gene expression profiles can provide useful information about their regulation and function. Therefore, to gain an insight into the potential functional differences of the members of the *N. benthamiana* families of Toc and Tom receptors, we analyzed their relative expression by RT-qPCR in photosynthetic (leaves and stems) and non-photosynthetic (roots) tissues at different weeks after germination (2, 4 and 6). Total RNAs extracted from a mix of leaves, stems and roots was used as reference sample. Expression levels of the *NbTOC34* gene showed practically uniform levels of relative expression in all tested tissues and stages (2W: p=0.053 and F=3.95; 4W: p=0.381 and F=1.136; 6W:p=0.208 and F=1.90) (**Figure 6A**). In contrast, the highest expression of the four Toc159 family members was generally observed in leaves, while stem and roots displayed less but similar relative levels of transcripts (**Figures 6B–E**). The largest difference between the levels of transcripts in leaves and stem/root tissues was observed with *NbTOC90*, two weeks after germination, since transcripts were nearly undetectable in stems and roots (p<0.0001 and F=139.5). This difference became increasingly smaller at four (p<0.0001 and F=41.46) and six (p=0.143 and F=6.675) weeks after germination not only in this gene but also in *NbTOC120* (2W: p=0.013 and F=6.97; 4W: p=0.051 and F=4.031; 6W:p=0.136 and F=2.475) (**Figure 6C**) and *NbTOC159B* (2W: p=0.048 and F=4.14; 4W: p=0.046 and F=9.87; 6W:p=0.056 and F=3.86) (**Figure 6B**) but, contrastingly, it became larger in *NbTOC159A* (2W: p=0.011 and F=15.28; 4W: p=0.0002 and F=24.75; 6W:p=0.003 and F=21.64) (**Figure 6D**).

Concerning mitochondrial receptors, the relative expression of *NbOM64* gene 2 weeks after germination was similar in all tissues (p=0.713; F=0.47) but its expression in roots relative to leaves and stems significantly decreased at four (p=0.0167, F=6.309; p_leaf vs root_= 0.0169) and six (p=0.0028, F=11.54; p_control vs root_= 0.00108; p_leaf vs root_= 0.0035; p_stem vs root_= 0.0068) weeks after germination (**Figure 6F**). Although there was not a significant difference in the relative expression of *NbTOM20.1* in different tissues and stages (2W: p=0.202 and F=1.94; 4W: p=0.141 and F=2.41; 6W:p=0.177 and F=2.11), it seems that there is a tendency towards higher relative expression in photosynthetic tissues than in roots (**Figure 6G**). This trend seems to be inverted at least two and four weeks after germination in *NbTOM20.2* (2W: p=0.383 and F=1.16; 4W: p=0.0005 and F=18.97; 6W:p=0.027 and F=5.27) (**Figure 6H**).

### Interaction of NbToc34 with the members of the Toc159 family in *N. benthamiana*

**Figure 7:**
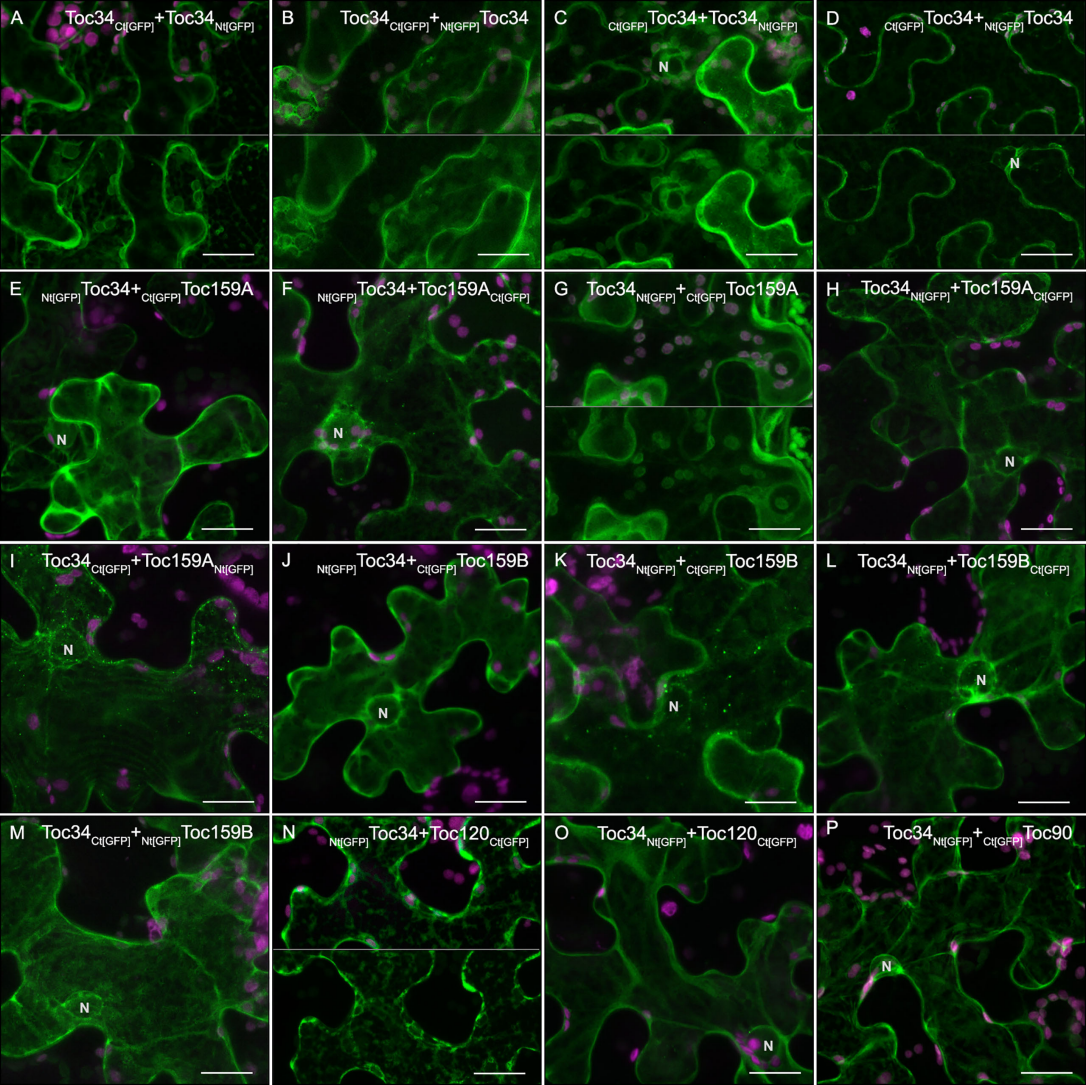
Bimolecular fluorescence complementation assay in leaf epidermal cells of *N. benthamiana*. Leaves were cotransfected with Agrobacterium carrying constructs for expression of full-length NbToc34 and either NbToc34, NbToc159A, NbToc159B, NbToc120 or NbToc90 tagged with Nt[GFP] or Ct[GFP] as indicated in the top of each panel. Only combinations that resulted in fluorescence visualization (positive interaction) by LSCM are displayed. Images of NbToc34 homodimerization **(A–D)**, as well as its interaction with NbToc159A **(E–I)**, NbToc159B **(J–M)**, NbToc120 **(N–O)** and NbToc90 **(P)**, are shown. All images correspond to Z-stack projections taken two days after infiltration. Chlorophyll fluorescence is shown in magenta. N indicates the position of the nucleus. Scale bars correspond to 20 µm.

Biochemical studies performed in *A. thaliana* have shown that a complex network of interactions can occur between the members of the Toc34 family and those of the Toc159 family through its GTPase domains. Particularly, AtToc159 preferentially associates with atToc33, while AtToc120 and AtToc132 especially interact with AtToc34, which in the absence of client proteins, also form homodimers. In addition, genetic and molecular studies have revealed that the acidic domain of the Toc receptors interacts with transit peptides of different client proteins. Therefore, structurally diverse Toc complexes can be assembled depending on the identity and relative abundance of the translocon receptors showing different abilities for preprotein recognition and translocation (Dutta et al., 2014; Schnell, 2019). To explore this possibility in *N. benthamiana*, we carried out bimolecular fluorescence complementation (BiFC) and yeast two-hybrid (Y2H) assays. Briefly, we fused each of the GFP fragments (see Materials and Methods section) to the amino and carboxyl terminus of the bait (NbToc34) and prey proteins (NbToc90, NbToc120, NbToc159A and NbToc159B) to generate Nt [YFP]-Bait/Prey, Ct [YFP]-Bait/Prey, Bait/Prey-Nt[YFP] and Bait/Prey-Ct[YFP] recombinant proteins. We screened all eight combinations for fluorescence complementation by LSCM: Bait-Ct[YFP]+Nt[YFP]-Prey, Ct[YFP]-Bait+Nt[YFP]-Prey, Bait-Nt[YFP]+Ct[YFP]-Prey, Nt[YFP]-Bait+Ct[YFP]-Prey, Bait-Ct[YFP]+Prey-Nt[YFP], Ct[YFP]-Bait+Prey-Nt[YFP], Bait-Nt[YFP]+Prey-Ct[YFP] and Nt[YFP]-Bait+ Prey-Ct[YFP] (**Supplementary Table S4**). Two days after infiltration, the fluorescent signal was detected in the combinations shown in **Figure 7** but not with the other options and negative controls (**Supplementary Table S4** and **Supplementary Figure S7**). The GFP fluorescence resulting from NbToc34 homodimerization was consistently observed in the cytoplasm and chloroplast surfaces in three out of four combinations (**Figures 7A–C**) and, occasionally, in _Ct[GFP]_Toc34+_Nt[GFP]_Toc34 (**Figure 7D**). Interestingly, the number of positive combinations including NbToc34 and NbToc90/120/159A/159B were dependent on each receptor, with NbToc159A showing the highest number of them (5/8, **Figures 7E–I**), followed by NbToc159B (4/8, **Figures 7J–M**), NbToc120 (3/8, **Figures 7N, O**) and NbToc90 (1/8, **Figure 7P**). In contrast to that observed with NbToc34 homodimerization, only two positive combinations were show in cytoplasm and chloroplast envelope (**Figures 7G–N**) while fluorescence in the rest of positive interactions was mainly located at the cytoplasm with occasional punctate structures but not in the nucleus. At least for AtToc159, it was shown that a cytoplasmic pool is active for interaction with AtToc33 lacking the membrane anchoring domain (Hiltbrunner et al., 2001) and exhibits specific transit peptide binding (Smith et al., 2004). Therefore, the visualization of receptor interactions in the cytoplasm is possible.

**Figure 8:**
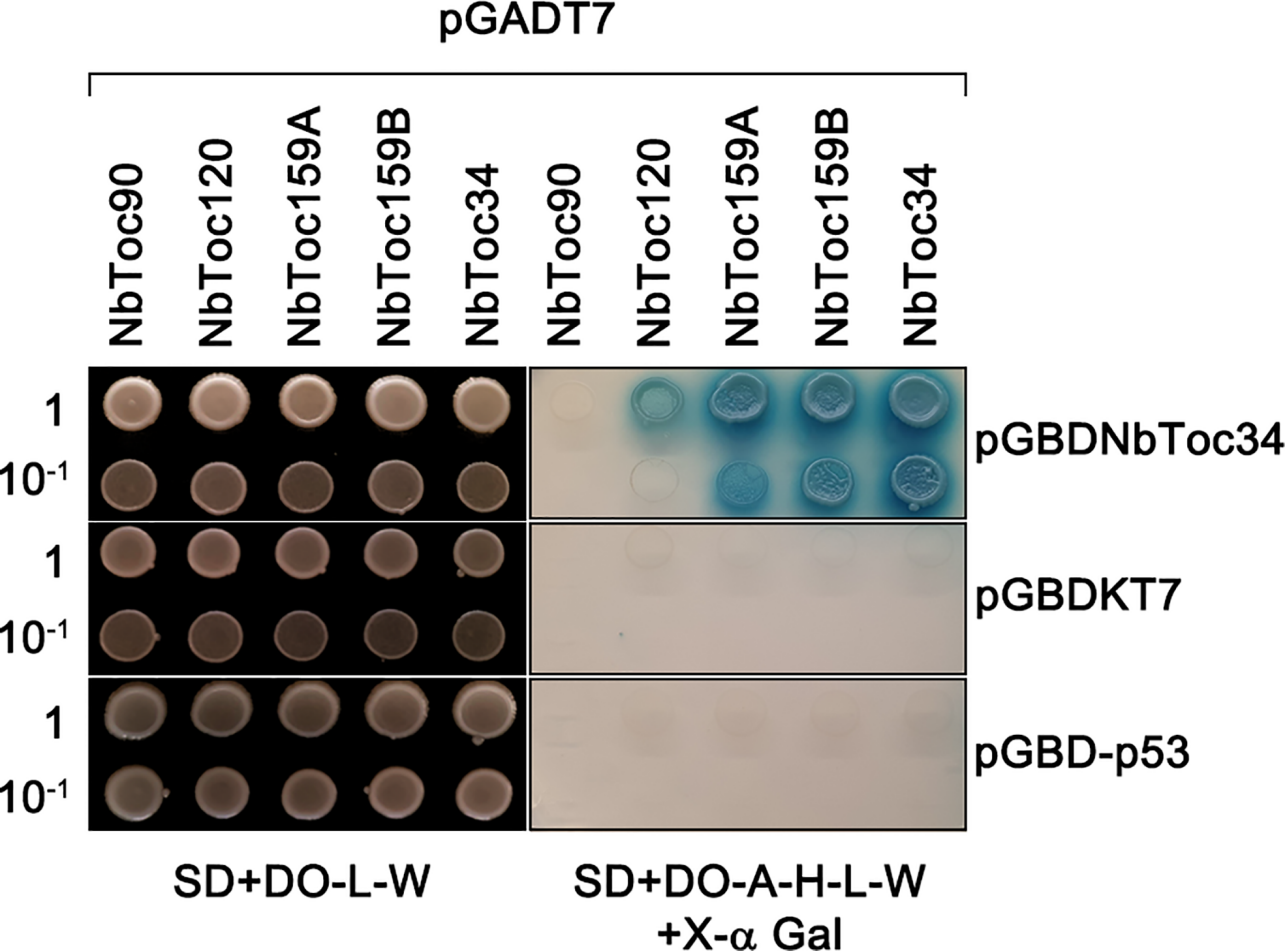
Yeast two-hybrid assay showing homodimerization of NbToc34 and its interaction with NbToc159A, NbToc159B and NbToc120. The cDNA of NbToc34 was fused to the GAL4 Binding Domain in pGADT to be used as bait. Besides, the cDNAs of NbToc34, NbToc159A, NbToc159B, NbToc120 and NbToc90 were fused to the GAL4 Activation Domain in pGBKT7 to be used as prey. The empty vector pGBKT7 and pGB-p53 holding a tumor suppressor were used as negative controls. Constructs were transformed into the yeast strain AH109 and double-plasmid transformants were selected by growing them in SD+DO-L-W medium. Double-plasmid transformants were then spot-plated in ten-fold serial dilutions in selective SD+DO-A-H-L-W+X-α Gal medium to detect the activation of HIS3, ADE2, and MEL1 reporter genes (right). A replicate was done in SD+DO-L-W medium (left) to assess equal plating of double-plasmid transformants yeast cells.

Next, we performed additional Y2H experiments to corroborate the above NbToc34 interactions. As negative controls, we used pGBDKT7 empty vector and pGBD-p53, a well-known tumor suppressor. The interactions were screened on the highly stringent SD-Ade-His-Leu-Trp+X-α-Gal (**Figure 8**, right) and replicates were made on nonselective SD-Leu-Trp plates to assess the growth of double-plasmid transformants (**Figure 8**, left). Growth was evident for NbToc34, NbToc159A, NbToc159B and NbToc120 baits, while no growth was observed among negative controls and NbToc90. Nevertheless, NbToc34 may interact more efficiently with NbToc34, NbToc159A and NbToc159B than NbToc120 as suggested by the comparatively faster growth, higher yield of cell mass and more intense blue staining (**Figure 8**). These results align with those obtained above in BiFC assays with NbToc159A and NbToc159B showing the highest number of positive combinations, and overall, indicating that NbToc34 could have an affinity higher for NbToc159A and NbToc159B than for NbToc120. Our findings also point to a lack of interaction between NbToc34 and NbToc90. Thus, differences in NbToc34 affinity could provide Toc complexes with a fine-tuning mechanism of preprotein recognition, which could depend on the availability and relative abundance of the Toc159 receptors.

## Discussion

Five million years ago, an ancient hybridization event occurred between members of sections *Noctiflorae* and *Sylvestres*, giving rise to the paleo-allotetraploid species *N. benthamiana*. Over the diploidization process, *N. benthamiana* has undergone extensive rearrangements between both subgenomes that only remain 19 chromosomes instead of 24, which is the sum of the parental chromosomes. Subgenome identification based on a phylogenomic approach revealed that genome downsizing mostly affected the paternally derived subgenome (50.3% of genes in NibSet-1 had a maternal origin, but only 34.4% had a paternal origin) (Schiavinato et al., 2020; Schiavinato, 2021). In this way, we found duplicated orthologs in *N. benthamiana* genome v1.0.1 predicted proteins and both NibSet-1 and NbDE protein datasets for many of the *A. thaliana* Toc and Tom core components. They were tentatively designed as NbToc34, NbToc90, NbToc120, NbToc159A, NbToc159B, NbTic22-III, NbTic22-IV, NbToc75-III, NbTom20-1, NbTom20-2, NbOm64, NbToc64 and NbTom40. We have assigned a maternal origin to each pair of *N. benthamiana* orthologs identified in this work, except for NbTic-III and NbTic-IV, which had a paternal origin. These findings, together with the gene structural resemblance observed between the duplicated genes, the fact that some of them exhibit different transcriptional orientations and DNA sequence identities were close to 100% indicates that duplicated locus comes from recent segmental duplications rather than be homeologs arising from ancient whole genome duplications.

Among the databases used, the NbDE dataset gave the most accurate functional annotation. However, some relevant information about the presence/absence of isoforms was still lacking. At least two Toc34 isoforms have been found in *A. thaliana*, maize, spinach and the moss *Physcomitrella patens* (Schwenkert et al., 2018). AtToc33 is the most abundant isoform that seems to be mostly in photosynthetic tissues, while AtToc34 is prominent in non-green tissues such as roots. Although AtToc34 and AtToc33 have different preferred precursor proteins, both paralogues show some functional redundancy. AtToc34 can complement AtToc33 knockout; only the double mutant is embryo-lethal (Constan et al., 2004; Hust and Gutensohn, 2006). However, the presence of multiple Toc34 isoforms showing different substrate specificity is not a general feature of plants. As occurs in pea and tomato, we only found one of them in *N. benthamiana*. The absence of redundant isoforms was also suggested by the strong albino phenotype observed for NbToc34-silenced plants and its homogeneous relative expression in roots, stems and leaves. In addition, our database and literature searches from other species in genera *Nicotiana* and *Solanum*, where genomic data are available, also identified a unique Toc34 homolog for each one, suggesting that this situation could be a common feature in the family *Solanaceae*.

The Toc159 family in *A. thaliana* is composed of four members, AtToc90/120/132/159. They differ in the length of their A domains, which may contribute to the transit peptide selectivity and, thus, to the functional specialization of the Toc159 receptors (Kubis et al., 2004). AtToc159 is highly expressed in young leaves and photosynthetic tissues and interacts preferentially with AtToc33. Together with AtToc75, they are assembled in Toc complexes that are important for photosynthetic preprotein import into leaf chloroplasts. AtToc159 knockout (*ppi2*) shows a severe albino phenotype that cannot grow beyond the cotyledon stage. AtToc90 is the less abundant receptor, but it also interacts with AtToc33 and partially restores *ppi2* mutant (Infanger et al., 2011). AtToc132 and AtToc120, which show a high degree of aa sequence identity (89.74% in G/M domains) and especially associate with AtToc34, are more abundant than AtToc159 in roots and may be more relevant for preprotein import into root leucoplasts (Ivanova et al., 2004). Single *toc132* and *toc120* mutants showed no visible phenotype, but *toc132/toc120* double homozygote resulted in a *toc159*-like bleached phenotype indicating a highly redundant functionality between them. Nevertheless, no functional overlap exists between AtToc120/132 and AtToc90/159 (Kubis et al., 2004). In this work, we also found four Toc159-related receptors in *N. benthamiana* but some differences with those of *A. thaliana* were evident. As in *A. thaliana*, NbToc90 lacked the A domain and was phylogenetically related to AtToc90 and other Toc90-annotated sequences from *Nicotiana* and *Solanum* sp. AtToc120/132 counterpart in *N. benthamiana* was a unique NbToc120 protein, while that of AtToc159A was phylogenetically defined by two orthologs, NbToc159A and NbToc159B. In contrast to *A. thaliana*, all three have a molecular weight of about 150 kDa and an A domain similar in length. This situation agrees with tomato, where up to four potential orthologs of AtToc159 (slToc159-1/4) were previously identified. Three of them (slToc159-1/2/4) were also around 150 kDa in size (Yan et al., 2014). slToc159-1 and slToc159-2 fell in the same clades that NbToc159B and NbToc159A, respectively, while slToc159-4 did in that of NbToc120. Our phylogenetic analysis suggested that this situation could be extended to other species belonging to the genera *Nicotiana* and *Solanum*, as we have stated above for NbToc34. The findings reported here also indicate that the degree of functional overlapping among Toc159-like receptors in *N. benthamiana* is higher than in *A. thaliana.* No recognizable phenotypes were observed for NbToc159-like receptor silenced plants, except for the pale one of *NbTOC159B*- and *NbTOC159A/B*-silenced plants, but albino leaf areas were observed after *NbTOC120/159A/159B* silencing. Finally, no differences in gene expression profiles between green and non-green tissues were detected for any NbToc159 receptor.

It has been reported that the specificity and fidelity of the nuclear-encoded plastid preprotein import are conferred by the initial and maybe simultaneous recognition of the transit peptides by both Toc159 and Toc34 families of receptors. Besides, AtToc34-GDP forms homodimers through interactions between the G domains in the absence of client proteins, but transit peptide binding promotes their dissociation, the GDP-GTP exchange and GTP hydrolysis (Paila et al., 2015; Schnell, 2019). We have shown that NbToc34 also interacts with itself and the three receptors with an acidic domain, NbToc159A, NbToc159B and NbToc120, indicating that NbToc34 and NbToc159 receptors may also combine to form different Toc complexes. However, more detailed biochemical and genetic studies are needed to determine whether these *N. benthamiana* Toc complexes have specific functional roles. In any case, *A. thaliana* Toc complexes, which either assemble Toc33/Toc159/90 or Toc34/Toc120/132, could be more efficient in recognizing a different subset of client preproteins than those of *N. benthamiana* because they are forced to share a single form of Toc34.

Tic22 is a hydrophilic protein located in the chloroplast intermembrane space and is the first component of Tic to interact with the transit peptide. Therefore, we also address its proper annotation and functional characterization in *N. benthamiana*. *A. thaliana* has two isoforms of Tic22, AtTic22-III and atTic22-IV, that define conserved clades in land plants (Kasmati et al., 2013). However, orthologs of AtTic22-III and IV in genera *Solanum* and *Nicotiana* found in NCBI search showed no differentiation between both isoforms and presented functional annotations such as Tic22-like, Tic22 and hypothetical proteins. Nevertheless, these sequences were equally distributed into two phylogenetical groups, each including either AtTicIII or AtTicIV, perhaps indicating that both isoforms are also present in the family *Solanaceae*. Silencing of NbTic22-III did not result in a visible phenotype either indicating functional redundancy or a regulatory rather than central role in Toc-Tic communication. In this way, neither the individual disruption of AtTic22-III and AtTic22-IV nor double-mutant affected *A. thaliana* development. The double mutant phenotype, consisting of reduced growth and photosynthetic performance, was visible only under high light conditions when high import rates of proteins were needed (Rudolf et al., 2013). A model was recently proposed in which Tic22 assists Toc75 POTRA domains in preprotein binding and chaperoning functions (O’Neil et al., 2017).

As happened in tomato (Paul et al., 2013), we could identify two AtTom20 orthologs in *N. benthamiana*, NbTom20-1 and NbTom20-2, but only one of AtOm64, which phylogenetically differed from AtToc64. Only NbDE dataset gives an annotation discerning between chloroplast and mitochondrial variants of the outer membrane protein 64. Regarding Tom20 receptors in *Nicotiana* sp., our phylogenetical analysis also revealed that most fell into two subgroups, each containing a tomato ortholog. These findings suggested that the number of Toc receptors and Tom receptors could be conserved among the species belonging to the genera *Nicotiana*. AtTom20-1 has not been identified as part of Tom complexes and its mRNA was rarely detected. Although some precursor recognition specificity has been assigned to the three remaining paralogues, only the quadruple mutant, disrupting AtTom20-2, AtTom20-3, AtTom20-4 and AtOm64 protein expression, causes embryo lethality (Lister et al., 2007; Duncan et al., 2013). This high functional redundancy was also supported by similar expression profiles of the four genes during development, except for a slight tendency of AtTom20-3 messenger to accumulate higher in roots than cotyledons and roots at 10 days post germination that was inverted in AtTom20-4. We observed a similar trend in the relative expression of *NbTOM20*-2 and *NbTOM20-1*/*NbOM64*, respectively. In addition, neither NbTom20-1, NbTom20-2 nor NbOm64-individually protein expression silenced plants displayed visible phenotypic abnormalities suggesting that Tom20 family members and Om64 in *N. benthamiana* as in *A. thaliana* are functionally redundant proteins. By contrast, silencing of either NbTom40 or NbToc75-III protein expression resulted in severe phenotypes indicating the absence of redundancy and their central role in protein translocation to mitochondria or chloroplast, respectively. Similarly, the corresponding null insertion mutants in *A. thaliana* showed an early embryo-lethal phenotype (Hust and Gutensohn, 2006; Hu et al., 2019).

Knowledge of the specific cell compartment where a protein localizes is a major determinant in genome annotation since it determines the range of functions that the protein may perform and could help to identify the potential interacting partners. Our results showed that the subcellular localization of *A. thaliana* and *N. benthamiana* orthologs produced identical fluorescent patterns. Toc receptors and Tic22-III were dually localized in the cytoplasm and chloroplast envelope, while Toc75-III was predominantly localized in the chloroplast surface. Due to the stability of the β-barrel scaffold, GFP from recombinant proteins can partially withstand proteasomal degradation leading to cytoplasmic fluorescence. Alternatively, cytoplasm localization is consistent with previous subcellular localization studies in *A. thaliana*, pea, and *Bienertia sinuspersici* by chloroplast fractionation, immunogold, immunofluorescence, and CLSM with GFP-tagged proteins; for example, 40% of the BsToc159 and BsToc132 receptors were found in the cytoplasm (Hiltbrunner et al., 2001; Lung and Chuong, 2012). At least for AtToc159, it was shown that the soluble form is active for interaction with AtToc33 lacking the membrane anchoring domain (Hiltbrunner et al., 2001) and exhibits specific transit peptide binding (Smith et al., 2004). Although the functional relevance of this soluble pool has been questioned (Becker et al., 2004), it was also proposed that Toc complex assembly is a dynamic process in which the soluble fraction of the receptors may help preproteins to reach the translocon (Bauer et al., 2002; Smith et al., 2002).

Although Tom40, Om64 and Tom20 are mitochondrial outer membrane proteins, they showed different fluorescent patterns, which most likely reflect the ways each associate with the outer membrane of the mitochondria or the pathways by which they reach the mitochondrial surface. Tom40 is a β-barrel protein that requires the Tom complex and the sorting and assembly machinery (SAM) for its insertion, while Om64 and Tom20 receptors are single-spanning proteins. Om64 is a signal-anchored protein with an N-terminal transmembrane domain. Although little information about the process is available, Om64 must be inserted into the outer membrane through the mitochondrial import machinery (MIM) with Hsp70 chaperones and their co-chaperones, the J-proteins, playing an essential role in the process (Wiedemann and Pfanner, 2017; Gupta and Becker, 2021). In our study, Om64 overexpression may disrupt mitochondrial membrane permeability causing morphological alterations that result in swollen mitochondria. Finally, Tom20 receptors are tail-anchored (TA) proteins that associate with the membrane through its C-terminally located transmembrane domain. Because of their topology, they require a posttranslational pathway to be inserted into the membrane, most likely involving Guided Entry of Tail-anchored (GET) proteins such as Get3 in yeast (Farkas et al., 2019). Get3 is a targeting factor that efficiently guides TA proteins to the ER but also interacts with some mitochondrial precursors. Moreover, when mitochondrial precursor accumulates in the cytoplasm, the GET pathway can direct them onto the ER surface from where they finally reach the mitochondria (Koch et al., 2021). Chloroplasts and mitochondria also contain proteins that are closely related to Get3. Three GET3 paralogs of *A. thaliana* were localized to the cytosol (AtGET3a), chloroplast (AtGET3b), and mitochondria (AtGET3c) (Xing et al., 2017; Mehlhorn et al., 2021). GET3b has already been involved in targeting TA proteins to the thylakoids (Anderson et al., 2021). Therefore, triple localization of NbTom20 and AtTom20 in the ER and mitochondrion and chloroplast envelope could be due to the oversaturation of the Get pathways.

To date, protein import systems in plants have been predominantly studied in *A. thaliana*, and a general picture of the molecular and regulation mechanisms will require studies in other species. Collectively, the findings presented are consistent with the notion that all proteins identified here are functional components of the chloroplast and mitochondrion protein import system in *N. benthamiana* and could likely be extended to other species in the genera *Nicotiana* and *Solanum*. Moreover, mitochondria and chloroplast are prime targets for viruses; in fact, many viral proteins involved in replication, movement, or plant defense overcoming are sent to these organelles. Knowing how these pathogen proteins hijack the plant import mechanisms to endosymbiotic organelles and identifying the specific host factors they use for that end will help to fight against them. Hence, we hope our results will be helpful for the further development of *N. benthamiana* as a research tool and will contribute not only to a better knowledge of organelle protein import mechanisms but also plant-pathogen relationships.

## Data availability statement

The datasets presented in this study can be found in online repositories. The names of the repository/repositories and accession number(s) can be found in the article/**Supplementary Material**.

## Author contributions

JN and VP conceived and designed the experiments. MS-B, AM-M and JN performed the experiments. JN and VP wrote the manuscript. All authors contributed to the article and approved the submitted version.

## Funding

This work was funded by grant PID2020-115571RB-I00 from the Spanish Agencia Estatal de Investigación (AEI) and Fondo Europeo de Desarrollo Regional (FEDER). JN and MS-B are the recipients of a postdoctoral contract and a PhD fellowship from the Ministerio de Ciencia, Innovación y Universidades of Spain, repectively.

## Acknowledgments

Thanks are due to L. Corachan-Valencia for her valuable technical assistance.

## Conflict of interest

The authors declare that the research was conducted in the absence of any commercial or financial relationships that could be construed as a potential conflict of interest.

## Publisher’s note

All claims expressed in this article are solely those of the authors and do not necessarily represent those of their affiliated organizations, or those of the publisher, the editors and the reviewers. Any product that may be evaluated in this article, or claim that may be made by its manufacturer, is not guaranteed or endorsed by the publisher.
