# Supplementary material for: Molecular characterization, targeting and expression analysis of chloroplast and mitochondrion protein import components in Nicotiana benthamiana
Source: Front Plant Sci. 2022 Oct 26;13:1040688. doi: 10.3389/fpls.2022.1040688 (PMC9643744; doi:10.3389/fpls.2022.1040688)
Supplement: Supplementary file 1 [file DataSheet_1.docx]

**Supplementary Figure Legends**

**Supplementary Figure S1.** Gene structure of the *N. benthamiana* core components of the Toc and Tom complexes, including *NbTIC22-III* and *NbTIC22-IV*. Filled orange and dark cyan boxes represent exons and UTR, respectively, and thin lines represent introns. The size of exons (E) is indicated in the direction from the 5’ to 3’ end of the gene. The scale line at the bottom indicates DNA length in kb pairs.

**Supplementary Figure S2.** Phylogenetic analysis of Toc receptors Toc34, Toc90, Toc120, Toc132 and Toc159 from *Arabidopsis thaliana/lyrata* (family *Brassicaceae*), *Cucumis sativus* (family *Cucurbitaceae*) and other species from *Nicotiana sp.* and *Solanum sp.* (family *Solanaceae*) were retrieved from NCBI and PhylomeDB databases (see accession numbers in Supplementary Table S2). The coat protein of the melon necrotic spot virus (GenBank: DQ339157.1) was used as an outgroup. The multiple sequence alignment was performed in MEGA XI using ClustalW. Evolutionary analysis was also conducted in MEGA XI by reconstructing the bootstrap consensus tree of sequences employing the Minimum Evolution method with 10000 bootstrap replicates. The bootstrap values with 10000 repetitions (%) are given at the respective nodes. All branches corresponding to partitions reproduced in less than 40% of bootstrap replicates were collapsed.

**Supplementary Figure S3.** Phylogenetic analysis of Tic receptors Tic22-III and Tic22IV from *Arabidopsis thaliana/lyrata* (family *Brassicaceae*) and other species from *Nicotiana sp*. and *Solanum sp*. (family *Solanaceae*). Sequences were retrieved from NCBI and PhylomeDB databases (see accession numbers in Figure and Supplementary Table S2). The coat protein of the melon necrotic spot virus (GenBank: DQ339157.1) was used as an outgroup. The multiple sequence alignment was performed in MEGA XI using ClustalW. Evolutionary analysis was also conducted in MEGA XI by reconstructing the bootstrap consensus tree of sequences employing the Minimum Evolution method with 10000 bootstrap replicates. The bootstrap values with 10000 repetitions (%) are given at the respective nodes. All branches corresponding to partitions reproduced in less than 40% of bootstrap replicates were collapsed.

**Supplementary Figure S4.** Phylogenetic analysis of Tom receptors Tom20 and Om64 from *Arabidopsis thaliana/lyrata* (family *Brassicaceae*), *Cucumis sativus* (family *Cucurbitaceae*) and other species from *Nicotiana sp.* and *Solanum sp.* (family *Solanaceae*) were retrieved from NCBI and PhylomeDB databases (see accession numbers in Supplementary Table S2). The coat protein of the melon necrotic spot virus (GenBank: DQ339157.1) was used as an outgroup. The multiple sequence alignment was performed in MEGA XI using ClustalW. Evolutionary analysis was also conducted in MEGA XI by reconstructing the bootstrap consensus tree of sequences employing the Minimum Evolution method with 10000 bootstrap replicates. The bootstrap values with 10000 repetitions (%) are given at the respective nodes. All branches corresponding to partitions reproduced in less than 40% of bootstrap replicates were collapsed.

**Supplementary Figure S5.** Phylogenetic analysis of Toc75-III and Tom40 from *Arabidopsis thaliana/lyrata* (family *Brassicaceae*) and other species from *Nicotiana sp.* and *Solanum sp.* (family *Solanaceae*) were retrieved from NCBI and PhylomeDB databases (see accession numbers in Figure and Supplementary Table S2). The coat protein of the melon necrotic spot virus (GenBank: DQ339157.1) was used as an outgroup. The multiple sequence alignment was performed in MEGA XI using ClustalW. Evolutionary analysis was also conducted in MEGA XI by reconstructing the bootstrap consensus tree of sequences employing the Minimum Evolution method with 10000 bootstrap replicates. The bootstrap values with 10000 repetitions (%) are given at the respective nodes. All branches corresponding to partitions reproduced in less than 40% of bootstrap replicates were collapsed.

**Supplementary Figure S6.** Subcellular localization of AtToc33-GFP (A), AtToc34-GFP (B), NbToc34-GFP (C), AtTic22-III-GFP (D), NbTic22-III-GFP (E), AtTom20-1-GFP (F), GFP-AtTom20-1 (G), AtTom20-2-GFP (H), GFP-AtTom20-1 (I), GFP-AtOm64 (J) and GFP-NbOm64 (K). GFP fusion proteins (green channel), indicated in the upper part of each panel, were expressed in epidermal cells of *N. benthamiana* by transient expression mediated by Agrobacterium. All LSCM images correspond to Z-stack projections taken two days after infiltration. Chlorophyll fluorescence is indicated (Chl) and shown in magenta. Scale bars correspond to 20 µm.

**Supplementary Figure S7.** Negative controls of constructs involved in positive combinations from the bimolecular fluorescence complementation assay showed in Figure 6. Leaves of *N. benthamiana* were cotransfected with Agrobacterium carrying the constructs for expression of either NbToc34, NbToc159A, NbToc159B, NbToc120, or NbToc90 tagged with Nt[GFP] or Ct[GFP] combined with free Nt[GFP] or Ct[GFP] as indicated in the top of each panel. All LSCM images correspond to Z-stack projections taken two days after infiltration. Chlorophyll fluorescence is shown in magenta. Scale bars correspond to 20 µm.
