# Supplementary figures and images for: Molecular characterization, targeting and expression analysis of chloroplast and mitochondrion protein import components in Nicotiana benthamiana
Source: Front Plant Sci. 2022 Oct 26;13:1040688. doi: 10.3389/fpls.2022.1040688 (PMC9643744; doi:10.3389/fpls.2022.1040688)

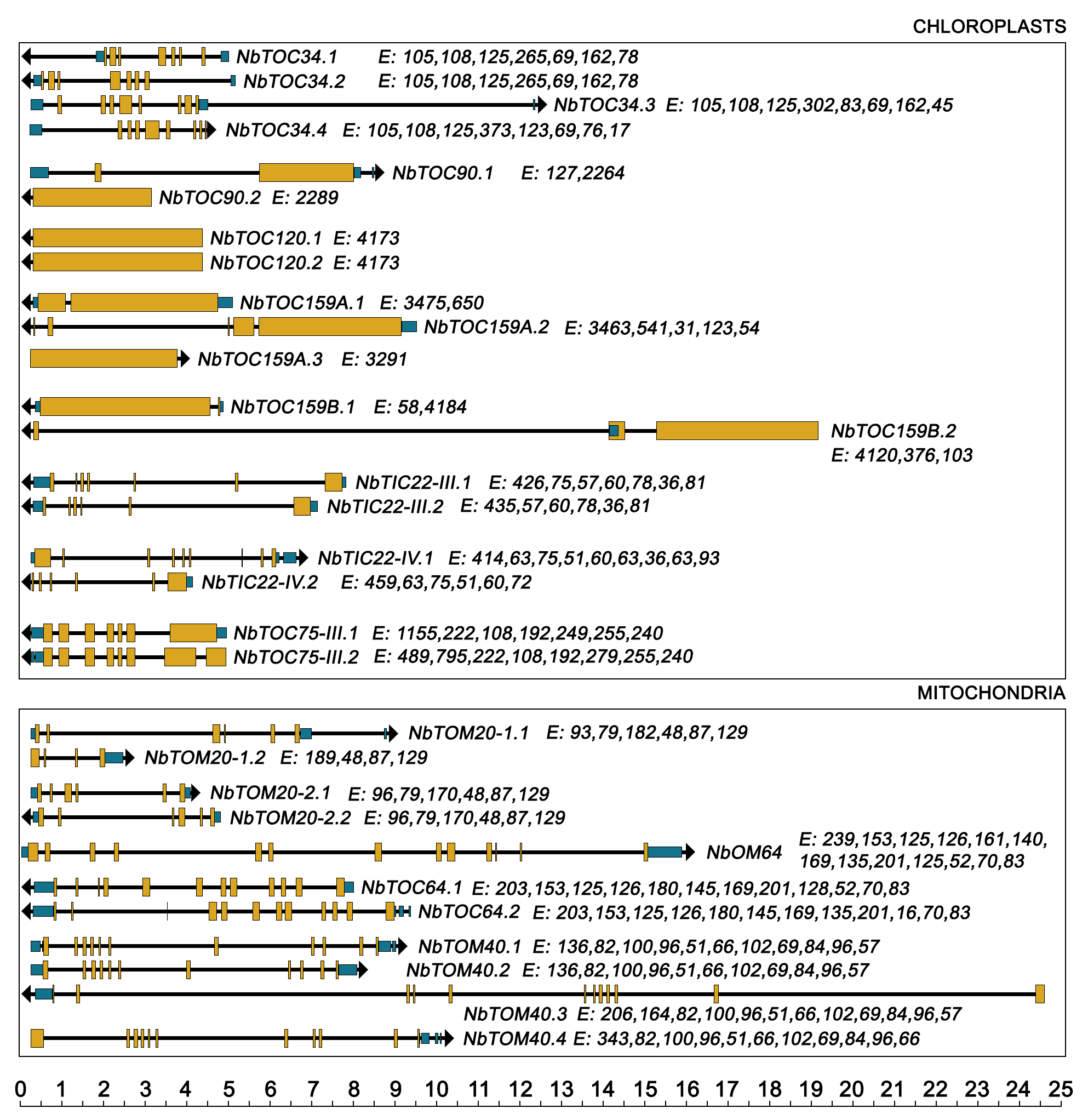

Supplement: Supplementary file 2 [file Image_1.tif]

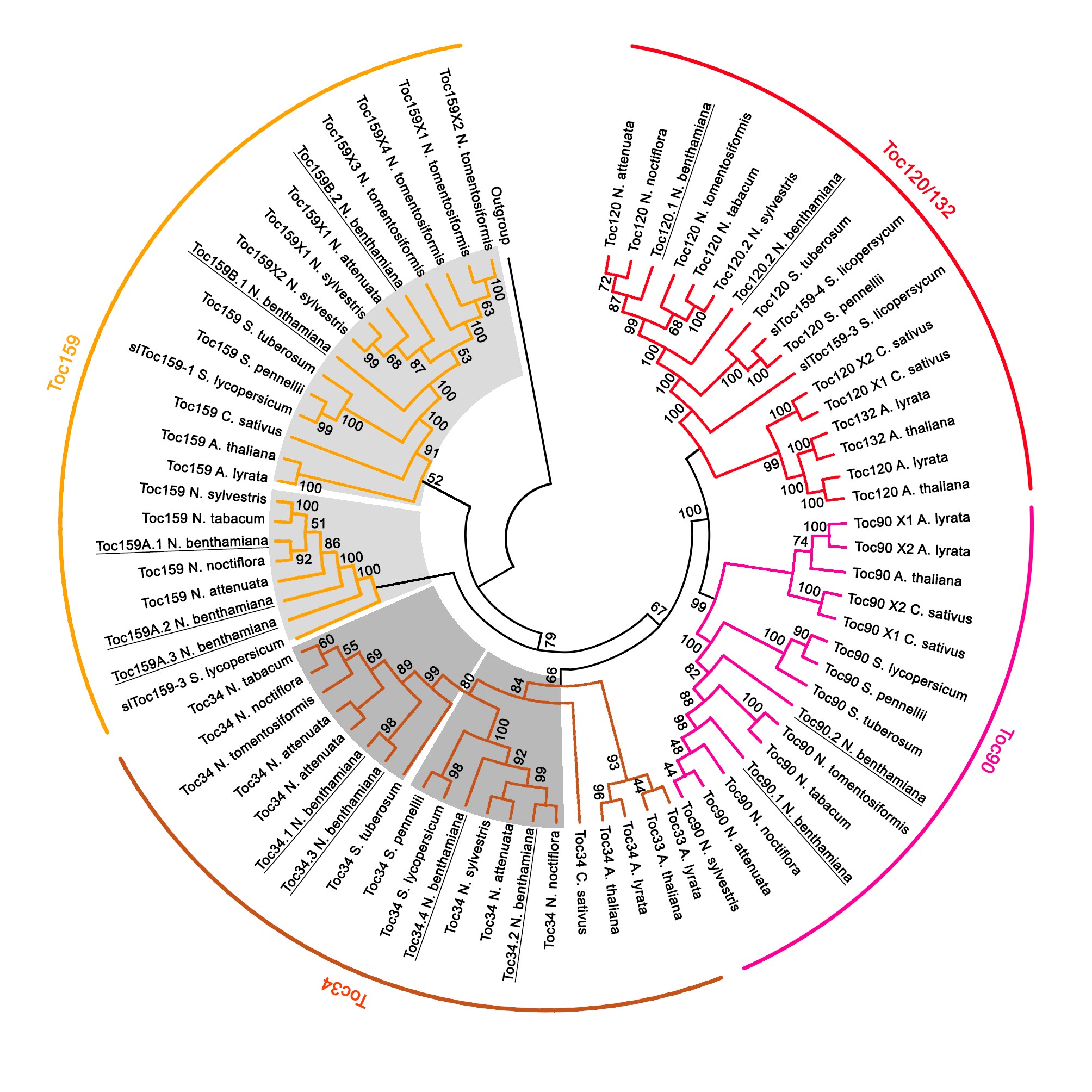

Supplement: Supplementary file 3 [file Image_2.tif]

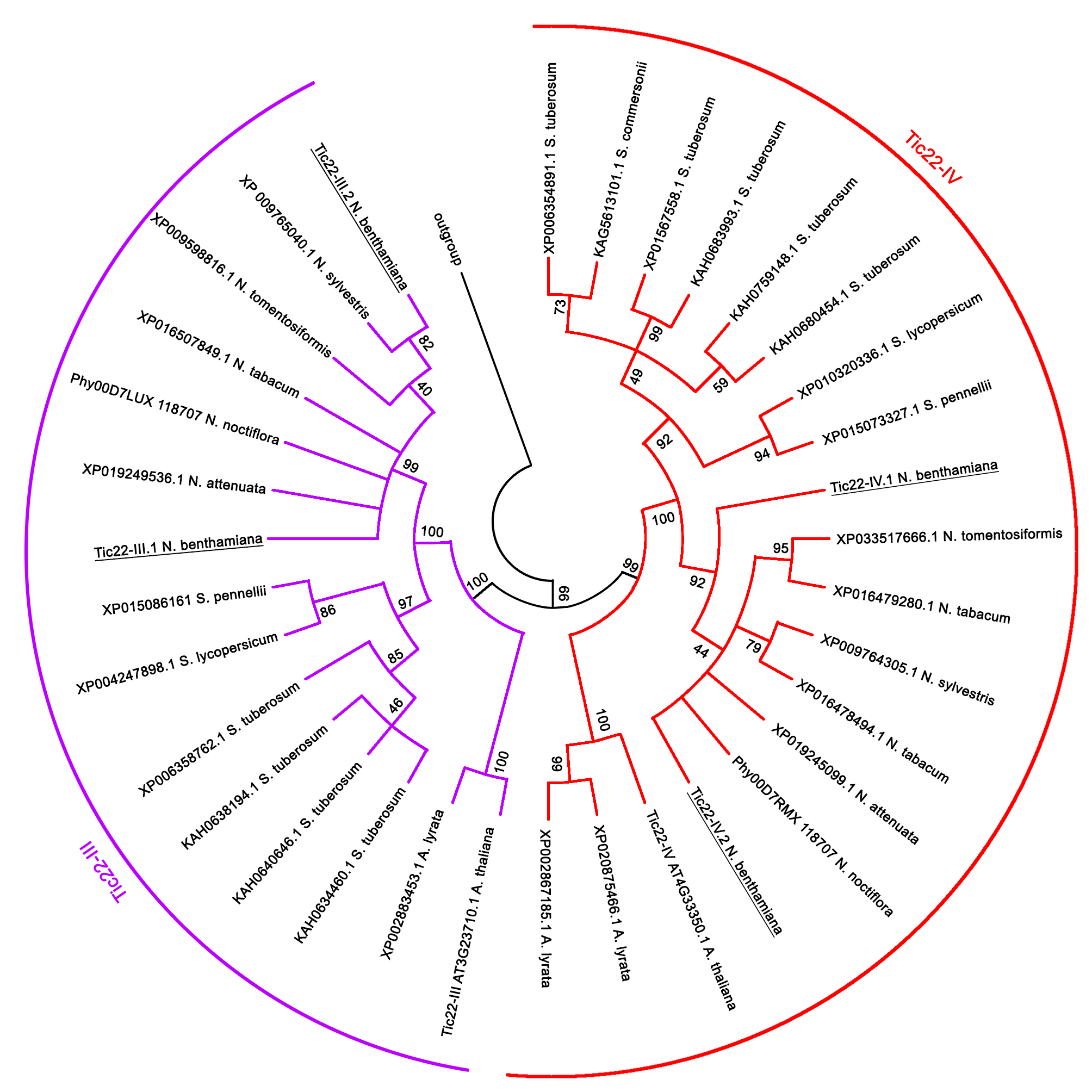

Supplement: Supplementary file 4 [file Image_3.tif]

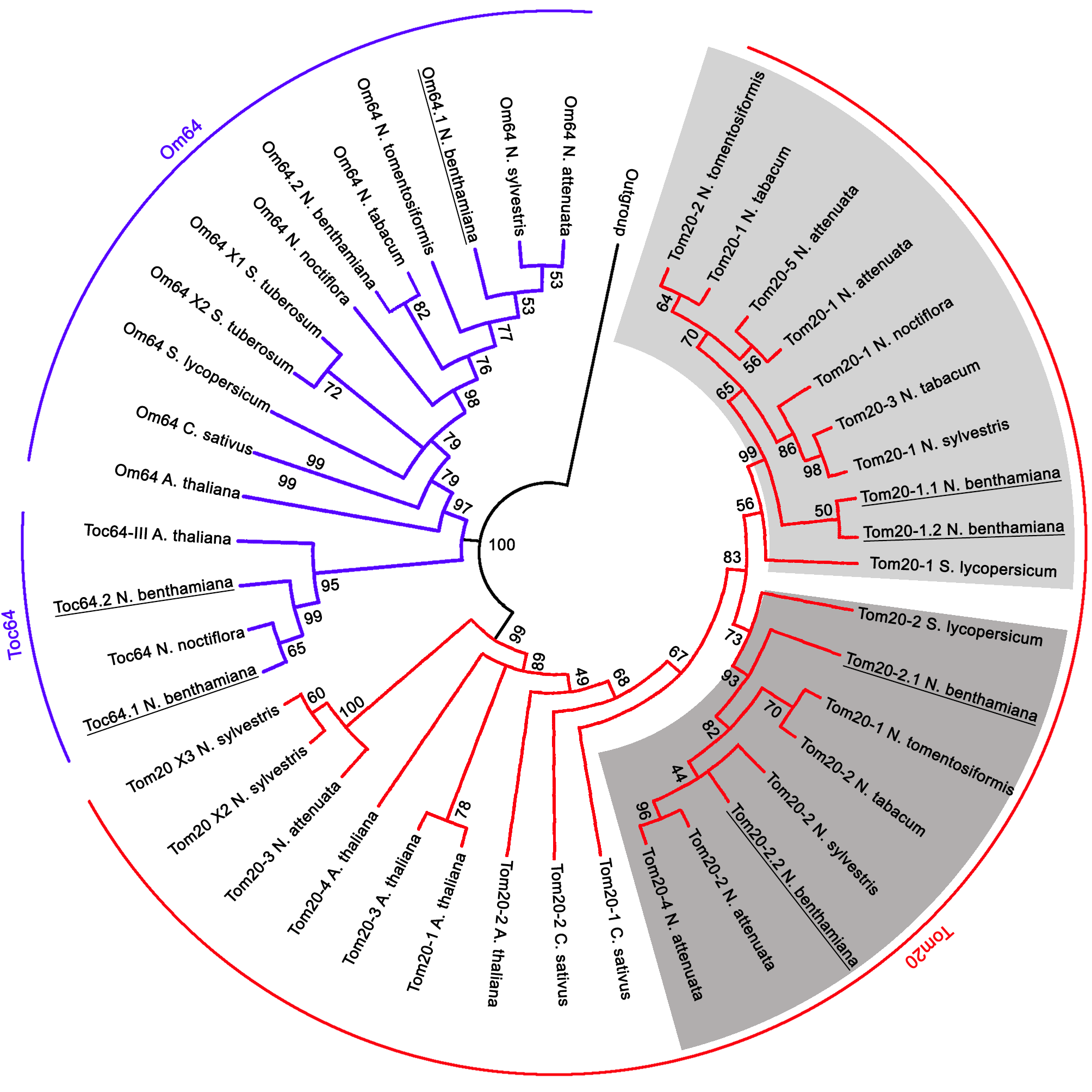

Supplement: Supplementary file 5 [file Image_4.tif]

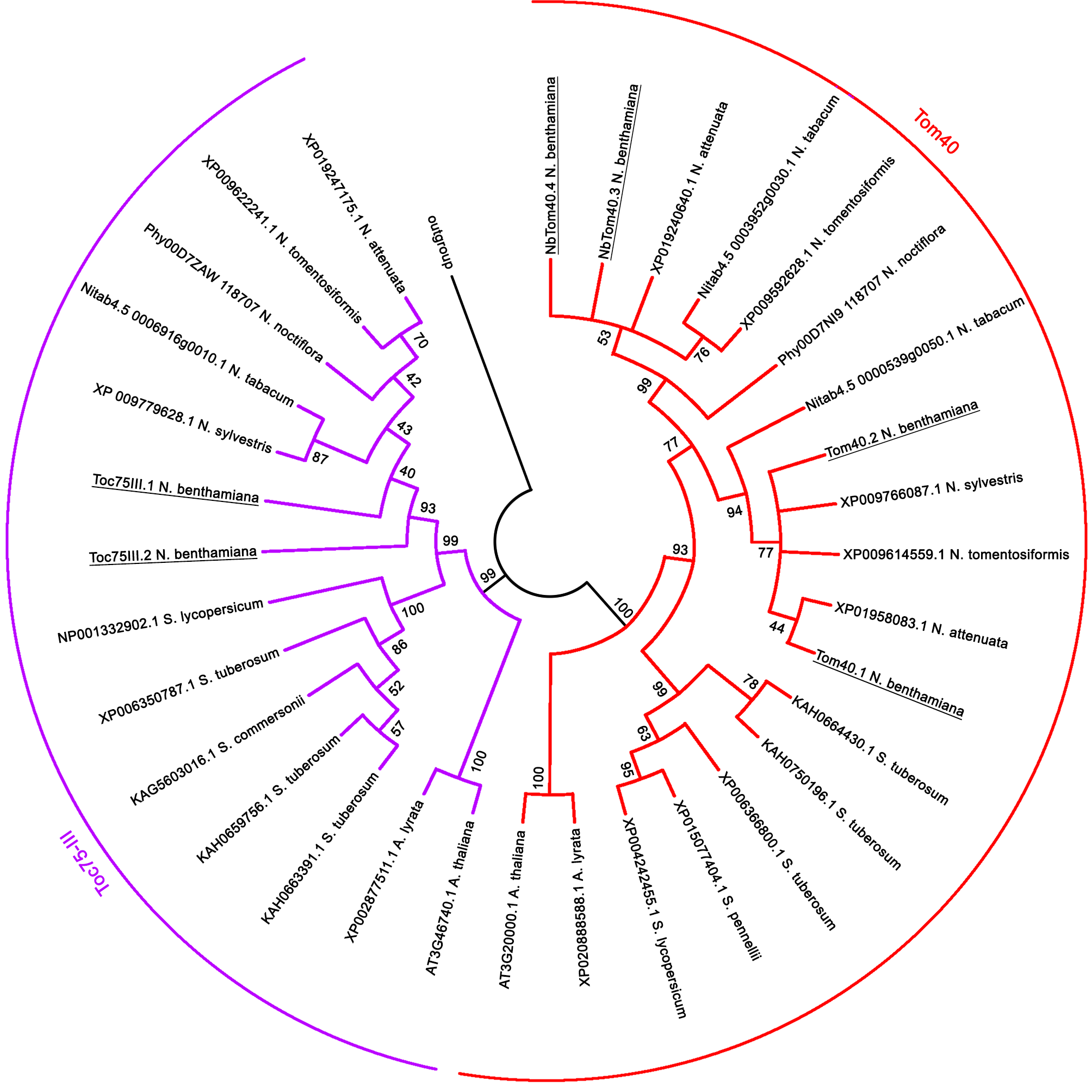

Supplement: Supplementary file 6 [file Image_5.tif]

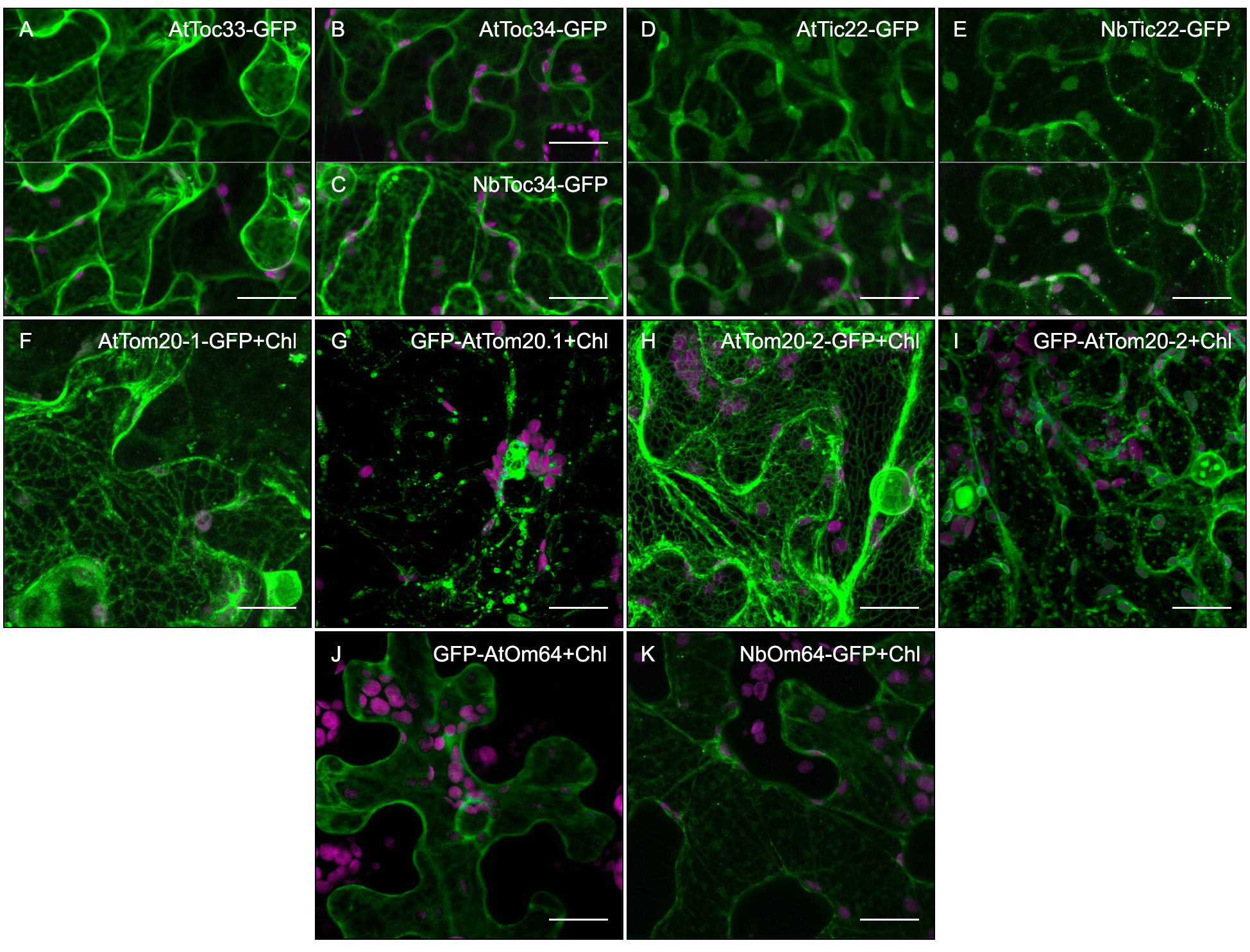

Supplement: Supplementary file 7 [file Image_6.tif]

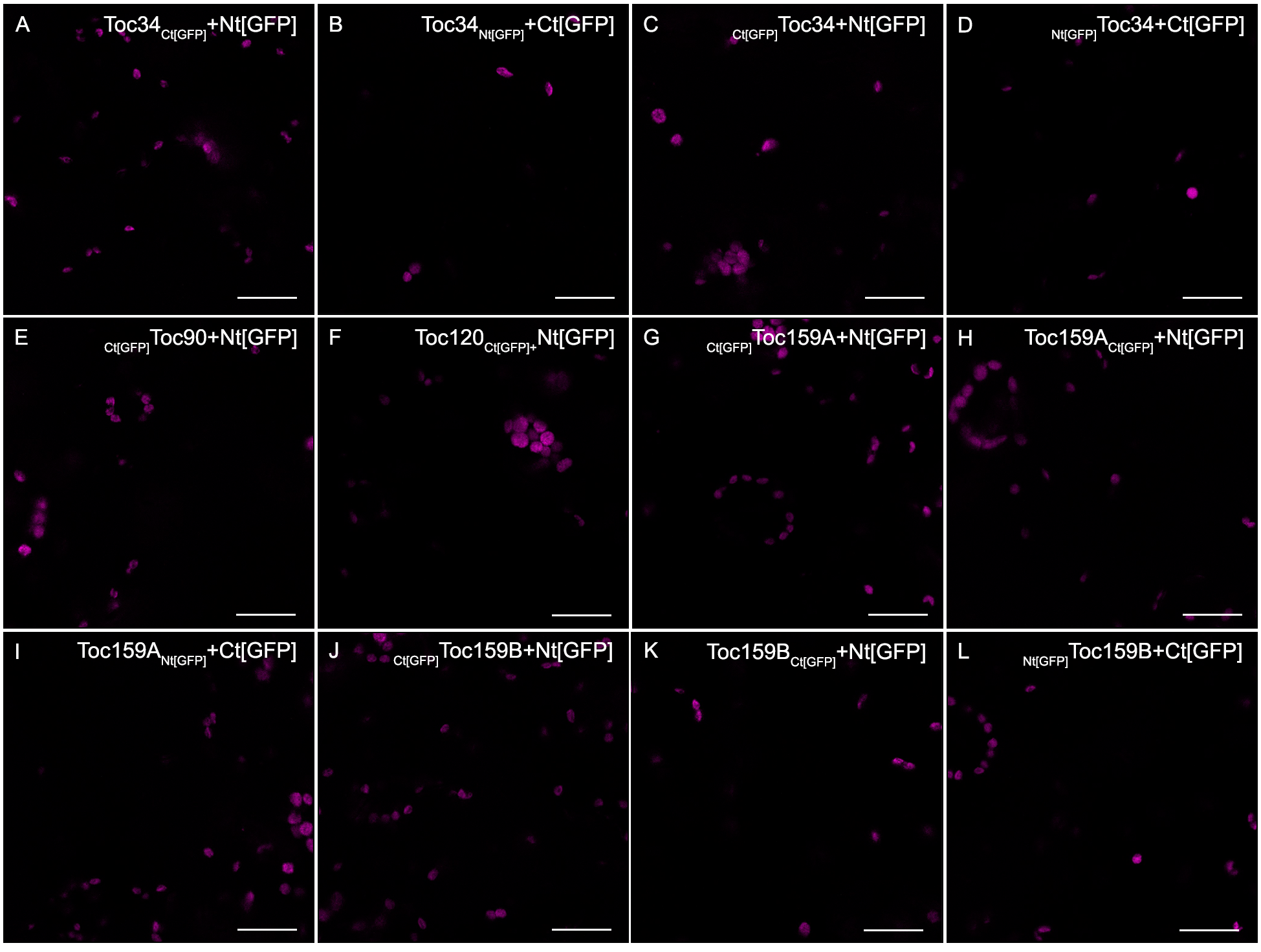

Supplement: Supplementary file 8 [file Image_7.tif]
