# Supplementary material for: Molecular characterization, targeting and expression analysis of chloroplast and mitochondrion protein import components in Nicotiana benthamiana
Source: Front Plant Sci. 2022 Oct 26;13:1040688. doi: 10.3389/fpls.2022.1040688 (PMC9643744; doi:10.3389/fpls.2022.1040688)
Supplement: Supplementary file 9 [file Table_1.docx]

| Supplementary Table S1. List of oligonucleotides used in this study. | | | |
| --- | --- | --- | --- |
| Subcellular localization and BiFC studies | | | |
| Plasmid | Gene | Forward primer | Reverse primer |
| pMOG800 | AtToc75-III | ACGTGGTCTCCCATGGCCGCCTTCTCCGTCAAC BsaI^1^ | ACGTGGTCTCGCTAGC**TTA**ATACCTCTCTCCAAATCGGAAG BsaI  ACGTGGTCTCGCTAGCATACCTCTCTCCAAATCGGAAG BsaI |
|  | AtToc33 | ACGTCCATGGGGTCTCTCGTTCGTGA NcoI | ACGTGCTAGC**CTA**AAGTGGCTTTCCACTTG NheI  ACGTGCTAGCAAGTGGCTTTCCACTTG NheI |
|  | AtToc34 | GCATCCATGGCAGCTTTGCAAACGCTT NcoI | ACGTGCTAGC**TCA**AGACCTTCGACTTGCTA  GCATGCTAGCAGACCTTCGACTTGCTAA NheI |
|  | AtToc90 | GCATTCATGAAAGGCTTCAAAGA PagI | GCATACTAGT**TTA**GGAAACGAGAAAA SpeI  GCATACTAGTGGAAACGAGAAAA SpeI |
|  | AtToc120 | AGCTCGTCTCTCATGGGAGATGGGGCTGAGATTG BsmBI | AGTCCGTCTCTCTAGGTGTCCATATTGCATTTG BsmBI  AGTCCGTCTCTCTAG**TCA**GTGTCCATATTGCATTTG BsmBI |
|  | AtToc132 | AGCTCGTCTCTCATGGGAGATGGGACTGAGTTTG BsmBI | AGCTCGTCTCTCTAGTTGTCCATATTGCGTTTG BsmBI  AGCTCGTCTCTCTAG**TCA**TTGTCCATATTGCGTTTG BsmBI |
|  | AtToc159 | ACGTGGTCTCGCATGGACTCAAAGTCGGTTAC BsaI | CATGGGTCTCGCTAGCGTACATGCTGTACTTGT BsaI  CATGGGTCTCGCTAG**TCA**CGTACATGCTGTACTTGT BsaI |
|  | AtTic22-III | ATGCGGTCTCTCATGAATTCAAACATTTTCCCACC BsaI | ATGCGGTCTCGCTAGCCTCCTGTGTTTGCTCAGTTG BsaI  ATGCGGTCTCGCTAG**TTA**CCTCCTGTGTTTGCTCAGTTG BsaI |
|  | AtTom40 | ACGTGGTCTCCCATGGCGGATCTTTTACCACCTC BsaI | ACGTGGTCTCGCTAGCACCAACTGTTAATCCGAAACC BsaI  ACGTGGTCTCGCTAGC**TTA**ACCAACTGTTAATCCGAAACC BsaI |
|  | AtTom20-1 | GCATCCATGGATAAGCTGAATTT NcoI | GCATTCTAGACCTTAGCTTTCGA XbaI  GCATTCTAGA**TCA**CCTTAGCTTTCGA XbaI |
|  | AtTom20-2 | ACGTCCATGGAGTTCTCTACCGCCG NcoI | ACGTGCTAGCTCTGGCAGGAGGTGGAGGG NheI  ACGTGCTAGC**TCA**TCTGGCAGGAGGTGGAGGG NheI |
|  | AtTom20-3 | ACGTCCATGGATACGGAAACTGAGTTC NcoI | ACGTGCTAGCACGAGGAGGAGAGACAGGC NheI  ACGTGCTAGC**CTA**ACGAGGAGGAGAGACAGGC NheI |
|  | AtTom20-4 | ACGTCCATGGATATGCAGAATGAAAACG NcoI | ACGTACTAGTCTGCCTTGACACCGGCGTC SpeI  ACGTACTAGT**TTA**CTGCCTTGACACCGGCGTC SpeI |
|  | AtOm64 | GCATCCATGGCCTCGAATACGCTTTCTTTG NcoI | ACGTGCTAGCTATGTGTTTTCGGAGTCTCTTC NheI  ACGTGCTAGC**TCA**TATGTGTTTTCGGAGTCTC NheI |
|  | NbToc75-III | ACGTCGTCTCCCATGGCGTCCATCGCCGCTCC BsmBI | ACGTCGTCTCGCTAGCGAATCTCTCTCCAAAACGG BsmBI  ACGTCGTCTCGCTAGC**CTA**GAATCTCTCTCCAAAACGG BsmBI |
|  | NbToc34 | AGTCCCATGGCATCTCAACTAATTAGAGA NcoI | AGTCGCTAGCTGCCCATGAAGGGCTGCTCT NheI  AGTCGCTAGC**TCA**TGCCCATGAAGGGCTGCTCT NheI |
|  | NbToc90 | AGCTCGTCTCTCATGATGAGTTTGAAGGATTGGG BsmBI | AGTCCGTCTCTCTAGCTCCCGCTTCCAGGGA BsmBI  AGTCCGTCTCTCTAGC**CTA**TCCCGCTTCCAGGGA BsmBI |
|  | NbToc120 | AGCTCGTCTCTCATGGAAAATGGGGAGGAAGTATT BsmBI | AGTCCGTCTCTCTAGAAATTGCACTGGTTGAGAGAAG  AGTCCGTCTCTCTAGA**TCA**AATTGCACTGGTTGAGAGAAG |
|  | NbToc159A | AGCTCGTCTCTCATGAATTCAAAGATTTATGGTGTTCCAC BsmBI | AGTCCGTCTCTCTAGCTATTAAGTTCTTTTCACTTGTTTGCG  AGTCCGTCTCTCTAGC**CTA**TATTAAGTTCTTTTCACTTGTTTGCG |
|  | NbToc159B | AGCTCGTCTCTCATGCTGAAGTCAGTGAAGC BsmBI | AGCTCGTCTCTCTAGCGTAGATCGAGTACTTCTCGC BsmBI  AGCTCGTCTCTCTAGC**TCA**GTAGATCGAGTACTTCTCGC BsmBI |
|  | NbTic22-III | ATGCGGTCTCTCATGAATATCTTCAAACCTAAACAGTC BsaI | AGTCGGTCTCGCTAGCCTTCTGGGAGTGATCTGTTGAA BsaI  AGTCGGTCTCGCTAGC**TCA**CTTCTGGGAGTGATCTGTTGAA BsaI |
|  | NbTom40 | ACGTCGTCTCCCATGGCCACTCTCATTCCTCC BsmBI | ACGTCGTCTCGCTAGCCTCTCCCACTGTAAGCCC BsmBI  ACGTCGTCTCGCTAGC**CTA**CTCTCCCACTGTAAGCCC BsmBI |
|  | NbTom20-1 | AGTCCCATGGAGCAAAACGATTTCGA NcoI | ATGCGCTAGCTCTTGGAGGAGGAGGAGGAGGCA NheI  ATGCGCTAGC**TTA**TCTTGGAGGAGGAGGAGGAGGCA NheI |
|  | NbTom20-2 | ACGTCCATGGATATGCAAAGCGAG NcoI | ACGTGCTAGCTTGGGTAGGAGGAGGGGGT NheI  ACGTGCTAGC**TTA**TTGGGTAGGAGGAGGGGGT NheI |
|  | NbOm64 | AGCTGGTCTCCCATGACAAAATTATCGAAGCTAAATG BsaI | ACGTGGTCTCGCTAGCACTAATTAGCTTTCTGAGTC BsaI  ACGTGGTCTCGCTAGC**TCA**ACTAATTAGCTTTCTGAGTC BsaI |
| Viral induced gene silencing (VIGS) | | | |
| Plasmid | Gene | Forward primer | Reverse primer |
| pDONR207  pTRV2 | GFP | *GGGGACAAGTTTGTACAAAAAAGCAGGCTTC*AGTAAAGGAGAAGAACTTTTC attB1^2^ | **CCTTGAAGAAGATGGTCCTCTC**^3^ |
|  | NbToc75-III | *GGGGACAAGTTTGTACAAAAAAGCAGGC*ACAATTGTTGGTGAGAGGAATG attB1 | *GGGGACCACTTTGTACAAGAAAGCTGGGT*GCATCTCAGCAGCCAGCTCCAG attB2 |
|  | NbToc34 | **GAGAGGACCATCTTCTTCAAGG**GTCCCGAAGATCAGAGG | *GGGGACCACTTTGTACAAGAAAGCTGGGTG*TGGAATGCTAATATAAAAGGAATTA attB2 |
|  | NbToc90 | **GAGAGGACCATCTTCTTCAAGG**TGATGAGTTTGAAGG | *GGGGACCACTTTGTACAAGAAAGCTGGGTG*AGCTTCAATCTTTACCACTG attB2 |
|  | NbToc120 | **GAGAGGACCATCTTCTTCAAGG**CACAGAGGACTTGAGA | *GGGGACCACTTTGTACAAGAAAGCTGGGTG*TACCGGCTGGCACTTATTG attB2 |
|  | NbToc159A | **GAGAGGACCATCTTCTTCAAGG**CTTTGAATACTCTAA | *GGGGACCACTTTGTACAAGAAAGCTGGGTG*AGTCCCTCAATGGTATCTGC attB2 |
|  | NbToc159B | **GAGAGGACCATCTTCTTCAAGG**ACGACCAAGGCCAACA | *GGGGACCACTTTGTACAAGAAAGCTGGGTG*ATCAACAAGCAGAAAAATGTTTCTGT attB2 |
|  | NbTic22-III | **GAGAGGACCATCTTCTTCAAGG**AAGGAAAGGGAAAGGA | *GGGGACCACTTTGTACAAGAAAGCTGGGTG*ATCTGTTGAAACATCGAAAC attB2 |
|  | NbTom40 | *GGGGACAAGTTTGTACAAAAAAGCAGGC*AGAACTTTTTGAAGGATTGCGC attB1 | *GGGGACCACTTTGTACAAGAAAGCTGGGT*ACCATGCCATGCGACATGTGTG attB2 |
|  | NbTom20-1 | **GAGAGGACCATCTTCTTCAAGG**TTTACTTTGACAAATCAGCT | *GGGGACCACTTTGTACAAGAAAGCTGGGT*GGGAGGAGGAGGAGGCACATTAGATT attB2 |
|  | NbTom20-2 | **GAGAGGACCATCTTCTTCAAGG**ATTGGAATTATCACAGTTCC | *GGGGACCACTTTGTACAAGAAAGCTGGGT*GTTGTGGATTTCCAAGTGCAACTCTG attB2 |
|  | NbOm64 | **GAGAGGACCATCTTCTTCAAGG**GGAAATGCTGCATATAAGG | *GGGGACCACTTTGTACAAGAAAGCTGGGT*GAGCCAAACTGGCAACCTTATT attB2 |
| Yeast2hybrid (Y2H) studies | | | |
| Plasmid | Gene | Forward primer | Reverse primer |
| pGBKT7/  pGADT7 | NbToc34 | ACGTGGTCTCGAATTCATGGCATCTCAACTAATTAGAGA BsaI | ACGTGGTCTCGGATCCCTATGCCCATGAAGGGCTGCTCT BsaI |
|  | NbToc90 | ACGTCGTCTCGAATTCATGATGAGTTTGAAGGATTGGG BsmBI | ACGTCGTCTCGGATCCTCATCCCGCTTCCAGGGA BsmBI |
|  | NbToc120 | ACGTCGTCTCGAATTCATGCTGAAGTCAGTGAAGC BsmBI | ACGTCGTCTCGGATCCTTAGTAGATCGAGTACTTCTCGC BsmBI |
|  | NbToc159A | ACGTCGTCTCGAATTCATGGAAAATGGGGAGGAAGTATT BsmBI | ACGTCGTCTCGGATCCTCAAAATTGCACTGGTTGAGAGAAG BsmBI |
|  | NbToc159B | ACGTCGTCTCGAATTCATGAATTCAAAGATTTATGGTGTTCC BsmBI | ACGTCGTCTCGGATCCCTATATTAAGTTCTTTTCACTTGTTTGCG BsmBI |
| Real Time RT-qPCR | | | |
| Gene | Access | Forward primer | Reverse primer |
| NbToc75-III | Niben101Scf01482g01002.1  Nbv5.1tr6393264 | CGCTCCTGGTATCGCACTTT | GATGAGGGTTTGGGGTTTTG |
| NbToc34 | Niben101Scf04926g06003.1  Nbv6.1trP36581 | CACCGAGGAATTGTGGTTCT | AATTGCAGCAGCCTGAATCT |
| NbToc90 | Niben101Scf09929g01001.1  Nbv6.1trP76192 | CTGGTTTATTGCCTCCCTCA | GCTTCAACAAGGGGAAATCA |
| NbToc120 | Niben101Scf04223g01022.1  Nbv6.1trP64107 | TGAGGACATGGTGGAACAAA | CTTTTCAAAGGCTGGAGTGC |
| NbToc159A | Niben101Scf06776g00001.1  Nbv6.1trP55981 | GATGGGGACAATCAGGCTTA | AGGAAACTGACCTGCAATGG |
| NbToc159B | Niben101Scf07086g00019.1  Nbv6.1trA113891 | CCATGGCTGGCTTTGATATT | CTTTAAGCCCGGTAACCACA |
| NbTic22-III | Niben101Scf28230g00015.1  Nbv6.1trP63905 | CACTTTTCCGCAACTCAACA | TGCATAAACAGGCACTCCAG |
| NbTom40 | Niben101Scf01291g00001.1  Nbv6.1trA47115 | AACTTGCCTTGCCCTATTCC | TGTGGGGCCCATAAGTACAC |
| NbTom20-1 | Niben101Scf00109g10030.1  Nbv6.1trA114286 | CAGGACCTTCGACATCAACA | ATCTTGGAGGAGGAGGAGGA |
| NbTom20-2 | Niben101Scf03766g01008.1  Nbv6.1trA6574 | TGCACTTGGAAATCCACAAA | CACCCATGCAACAATACCAA |
| NbOm64 | Niben101Scf02133g01001.1  Nbv6.1trP34412 | GCATCAAACGTACCCAGCTT | GTTTCACCGCTTTCATCCAT |
