# Supplementary material for: Molecular characterization, targeting and expression analysis of chloroplast and mitochondrion protein import components in Nicotiana benthamiana
Source: Front Plant Sci. 2022 Oct 26;13:1040688. doi: 10.3389/fpls.2022.1040688 (PMC9643744; doi:10.3389/fpls.2022.1040688)
Supplement: Supplementary file 10 [file Table_2.docx]

| Supplementary Table S2. Accession numbers of Tom and Toc receptors of different plant species used in phylogenetic analysis | | | | | | |
| --- | --- | --- | --- | --- | --- | --- |
| Species | Protein | Accession number | Species | Protein |  | Accession number |
| *A. lyrata* | Toc33 | XP_002892097.1 |  |  | |  |
|  | Toc34 | XP_020876012.1 |  |  | |  |
|  | Toc90 X1 | XP_020879152.1 |  |  | |  |
|  | Toc90 X2 | XP_002873990.1 |  |  | |  |
|  | Toc120 | XP_020889089.1 |  |  | |  |
|  | Toc132 | XP_020887996.1 |  |  | |  |
|  | Toc159 | XP_002874910.1 |  |  | |  |
| *A. thaliana* | Toc33 | AT1G02280.1 | ***A. thaliana*** | Tom20-1 | | AT3G27070.1 |
|  | Toc34 | AT5G05000.1 |  | Tom20-2 | | AT1G27390.1 |
|  | Toc90 | AT5G20300.1 |  | Tom20-3 | | AT3G27080.1 |
|  | Toc120 | AT3G16620.1 |  | Tom20-4 | | AT5G40930.1 |
|  | Toc132 | AT2G16640.1 |  | Om64 | | AT5G09420.1 |
|  | Toc159 | AT4G02510.1 |  |  |  |  |
| *N. tomentosiformis* | Toc34 | XP_009599441.1 | ***N. tomentosiformis*** | Tom20-1 | | XP_009601172.1 |
|  | Toc90 | XP_009608571.1 |  | Tom20-2 | | XP_009620342.1 |
|  | Toc120 | XP_009610657.1 |  | Om64 | | XP_009624799.1 |
|  | Toc159 X1  Toc159 X2  Toc159 X3  Toc159 X4 | XP_009602186.1  XP_018626732.1  XP_018626733.1  XP_018626735.1 |  |  |  |  |
| *N. attenuata* | Toc34.1  Toc34.2 | XP_019231210.1  XP_019245357.1 | ***N. attenuata*** | Tom20-1 | | XP_019226716.1 |
|  | Toc90 | XP_019255958.1 |  | Tom20-2 | | XP_019239067.1 |
|  | Toc120.1  Toc120.2 | XP_019253520.1  XP_019255958.1 |  | Tom20-3 | | OIT29074.1 |
|  |  |  |  | Tom20-4 | | XP_019239067.1 |
|  | Toc159 | XP_019241310.1 |  | Tom20-5 | | XP_019226716.1 |
|  | Toc159 | XP_019232171.1 |  | Om64 | | XP_019249941.1 |
| *N. tabacum* | Toc34 | XP_016457516.1 | ***N. tabacum*** | Tom20-1 | | XP_016457148.1 |
|  | Toc90 | XP_016508006.1 |  | Tom20-2 | | XP_016438094.1 |
|  | Toc120 | XP_016485376.1 |  | Tom20-3 | | XP_016487017.1 |
|  | Toc159 | XP_009769991.1 |  | Om64 | | XP_016456544.1 |
| *N. sylvestris* | Toc34 | XP_009779337.1 | ***N. sylvestris*** | Tom20-1 | | XP_009790058.1 |
|  | Toc90 | XP_009791876.1 |  | Tom20-2 | | XP_009783275.1 |
|  | Toc120 | XP_009768002.1 |  | Tom20 X2 | | XP_009785207.1 |
|  | Toc159  Toc159 X1  Toc159 X2 | XP_009769991.1  XP_009781898.1  XP_009781899.1 |  | Tom20 X3 | | XP_009785208.1 |
|  |  |  |  | Om64 | | XP_009776046.1 |
| *N. noctiflora* | Toc 34 | Phy00D7QQR_118707 | ***N. noctiflora*** | Tom20.1 | | Phy00D8263_118707 |
|  | Toc 90 | Phy00D7QME_118707 |  |  |  |  |
|  | Toc 120 | Phy00D7YSH_118707 |  |  |  |  |
|  | Toc 159 | Phy00D830G_118707 |  | Om64 | | Phy00D80ZF_118707 |
| *S. lycopersicum* | Toc34 | XP_004239977.2 |  |  |  |  |
|  | Toc90 | XP_004242739.1 | ***S. lycopersicum*** | Tom20-1 | | XP_004231778.1 |
|  | Toc120 | XP_004231012.1 |  | Tom20-2 | | XP_004233293.1 |
|  | Toc159 | XP_010326580.1 |  | Om64 | | XP_004248799.1 |
| *S. tuberosum* | Toc 34 | XP_006358174.1 | ***S. tuberosum*** | Om64 X1 | | XP_006341842.1 |
|  | Toc 90 | XP_006359492.1 |  |  |  |  |
|  | Toc 120 | XP_006359664.1 |  | Om64 X2 | | XP_015161806.1 |
|  | Toc 159 | XP_015169888.1 |  |  |  |  |
| *S. pennellii* | Toc 34 | XP_015074809.1 |  |  | |  |
|  | Toc 90 | XP_015087308.1 |  |  |  |  |
|  | Toc 120 | XP_015089777.1 |  |  | |  |
|  | Toc 159 | XP_015082300.1 |  |  | |  |
| *C. sativus* | Toc 34 | XP_004146141.1 | ***C. sativus*** | Tom20-1 | | XP_004142933.1 |
|  | Toc 90 X1  Toc 90 X2 | XP_011648710.2  XP_011648711.1 |  | Tom20-2 | | XP_004145663.1 |
|  | Toc 120 X1  Toc 120 X2 | XP_004144917.2  XP_031745274.1 |  | Om64 | | XP_004136877.1 |
|  | Toc 159 | XP_004152365.2 |  |  |  |  |
