# Supplementary material for: Molecular characterization, targeting and expression analysis of chloroplast and mitochondrion protein import components in Nicotiana benthamiana
Source: Front Plant Sci. 2022 Oct 26;13:1040688. doi: 10.3389/fpls.2022.1040688 (PMC9643744; doi:10.3389/fpls.2022.1040688)
Supplement: Supplementary file 11 [file Table_3.docx]

| Supplementary Table S3. List of Nicotiana benthamiana accession numbers of putative Tom and Toc receptors, main pores and Tic22, assignment to the parental ancestor and amino acid sequence identity | | | | | |
| --- | --- | --- | --- | --- | --- |
| Protein | Accession number:  Sol Genomics Network  NibSet1-1  NbDE* | Scaffold | Parental origin | Chromosome  (NbLab330 database) | Amino acid identity |
| NbToc75-III.1 | Niben101Scf01482g01002.1  g13969.t1 (100%)  NbD011778.1 (100%) | Paternal | Maternal | 18 | Niben101Scf01482g01002.1 vs Niben101Scf08757g00006.1 = 92.2% |
| NbToc75-III.2 | Niben101Scf08757g00006.1  g35129.t1 (94.3%)  NbD043052.1 (99.63%) | Maternal | Maternal | 09 |  |
| NbToc34.1 | Niben101Scf04926g06003.1  g17921.t1 (98.01)  NbD001798.1 (99.66%) | Maternal | Maternal | 16 | Niben101Scf04926g06003.1 vs Niben101Scf00454g05025.1 = 85.4% |
| NbToc34.2 | Niben101Scf00454g05025.1  g24833.t1 (98.33%)  NbD004316.1 (100%) | Maternal | Maternal | 10 |  |
| NbToc34.3 | Niben101Scf00163g07005.1  g39689.t1 (100%) | Maternal | Maternal | 06 | Niben101Scf04926g06003.1 vs  Niben101Scf00163g07005.1 = 94.44% |
| NbToc34.4 | Niben101Scf08179g01017.1  g5776.t1 (99.02%) | Paternal | orphan | 15 | Niben101Scf08179g01017.1  vs Niben101Scf00454g05025.1 = 96.08% |
| NbToc90.1 | Niben101Scf09929g01001.1  g54947.t1 (100%)  NbD045210.1 (100%) | Maternal | Maternal | 16 | Niben101Scf09929g01001.1 vs Niben101Scf04918g02009.1 = 92.51% |
| NbToc90.2 | Niben101Scf04918g02009.1  g91899.t1 (97.9%)  NbD031159.1 (99.08%) | Maternal | Maternal | 07 |  |
| NbToc120.1 | Niben101Scf04223g01022.1  g65878.t1 (100%)  NbD028297.1 (100%) | Orphan | Maternal | 08 | Niben101Scf04223g01022.1 vs Niben101Scf01847g07003.1 = 93.52% |
| NbToc120.2 | Niben101Scf01847g07003.1  g36650.t1 (100%)  NbD014331.1 (100%) | Ø | Maternal | 18 |  |
| NbToc159A.1 | Niben101Scf06776g00001.1  g62550.t1 (100%)  NbD037689.1(100%) | Maternal | Maternal | 09 | Niben101Scf06776g00001.1 vs Niben101Scf03857g01008.1 = 90.32%  Niben101Scf06776g00001.1 vs Niben101Scf05044g00002.1= 92.81%%  Niben101Scf03857g01008.1 vs  Niben101Scf05044g00002.1= 95.08%% |
| NbToc159A.2 | Niben101Scf03857g01008.1  g23395.t1 (98.01%)  NbD026259.1(98.01%) | Maternal | Maternal | 04 |  |
| NbToc159A.3 | Niben101Scf05044g00002.1  g13934.t1  NbD031696.1 | Maternal | Maternal | 06 |  |
| NbToc159B.1 | Niben101Scf07086g00019.1  g92014.t1 (100%)  NbD038692.1 (100%) | Ø | Maternal | 18 | Niben101Scf07086g00019.1 vs Niben101Scf03648g02002.1 = 95.33% |
| NbToc159B.2 | Niben101Scf03648g02002.1  g71266.t1 (99.86%)  NbD025242.1(99.79%) | Maternal | Maternal | 08 |  |
| NbTic22-III.1 | Niben101Scf28230g00015.1  g42700.t1 (100%)  NbD052702.1 (100%)  NbD021651.1 (96.49%) | Paternal | Paternal | 18 | Niben101Scf28230g00015.1 vs  Niben101Scf02964g00008.1 = 95.16% |
| NbTic22-III.2 | Niben101Scf02964g00008.1  g16180.t1 (100%)  NbE05066886.1 (95.96%) | Orphan | Orphan | 08 |  |
| NbTic22-IV.1 | Niben101Scf13041g02009.1  g23894.t1 (100%)  NbD048958.1(99.64%)  NbD001113.1 (94.14%) | Paternal | Paternal | 03 | Niben101Scf00090g13017.1 vs  Niben101Scf13041g02009.1 = 93.00% |
| NbTic22-IV.2 | Niben101Scf00090g13017.1  g20888.t1 (99.6%) | Orphan | Paternal | 15 |  |
| NbTom40.1 | Niben101Scf01291g00001.1  g85929.t1 (100%)  NbD010422.1 (100%) | Maternal | Maternal | 19 | Niben101Scf01291g00001.1 vs Niben101Scf01451g05003.1 = 97.76% |
| NbTom40.2 | Niben101Scf01451g05003.1  g78581.t1 (100%)  NbD011590.1 (100%) | Maternal | Maternal | 05 |  |
| NbTom40.3 | Niben101Scf05971g00007.1  g18096.t1 (100%)  NbD035191.1 (100%) | Maternal | Maternal | 02 | Niben101Scf01291g00001.1 vs Niben101Scf05971g00007.1 =92.33%  Niben101Scf05971g00007.1 vs Niben101Scf04436g15042.1 = 98.17%  Niben101Scf05971g00007.1 vs Niben101Scf01451g05003.1 = 92.33%  Niben101Scf01291g00001.1 vs Niben101Scf04436g15042.1 = 92.33% |
| NbTom40.4 | Niben101Scf04436g15042.1  g29593.t1 (100%)  NbD029233.1 (100%) | Maternal | Maternal | 12 |  |
| NbTom20-1.1 | Niben101Scf00109g10030.1  g1115.t1 (100%)  NbD001253.1 (100%) | Ø | Maternal | 19 | Niben101Scf14939g01027.1vs Niben101Scf00109g10030.1 = 95.61% |
| NbTom20-1.2 | Niben101Scf14939g01027.1  g1115.t1 (100%)  NbD001253.1 (100%) | Parental | Maternal | 02 |  |
| NbTom20-2.1 | Niben101Scf03766g01008.1  g8578.t1 (100%)  NbD025768.1 (100%)  NbD031933.1 (95.04%) | Maternal | Maternal | 02 | Niben101Scf03766g01008.1 vs Niben101Scf05095g01004.1 = 94.36% |
| NbTom20-2.2 | Niben101Scf05095g01004.1  g56513.t1 (100%)  NbD050116 (100%) | Orphan | Maternal | 15 |  |
| NbOm64.1 | Niben101Scf02133g01001.1  g93882.t1 (99.83%)  NbD016248.1 (99.83%) | Paternal | Maternal | 15 | Niben101Scf02133g01001.1 vs  NbD010896.1 = 97.64% in shared region  Niben101Scf02133g01001.1 vs  Niben101Scf08675g00023.1 = 49.82%  Niben101Scf02133g01001.1 vs  Niben101Scf08653g07031.1 = 50.53 %  Niben101Scf08675g00023.1 vs  Niben101Scf08653g07031.1 = 95.87% |
| NbOm64.2 | NbD010896.1 |  |  | 16 |  |
| NbToc64-III.1 | Niben101Scf08675g00023.1  g49103.t1 (100%)  NbD042788.1 (99.63%) | Maternal | Maternal | 16 |  |
| NbToc64-III.2 | Niben101Scf08653g07031.1  g26195.t1 (98.96%)  NbD042739.1 (98.96%) | Maternal | Orphan | 6 |  |
| ** percentage of identity between the aa sequences retrieved from Sol Genomics Network and NibSet1-1/NbDE datasets* | | | | | |
