# Supplementary material for: Molecular characterization, targeting and expression analysis of chloroplast and mitochondrion protein import components in Nicotiana benthamiana
Source: Front Plant Sci. 2022 Oct 26;13:1040688. doi: 10.3389/fpls.2022.1040688 (PMC9643744; doi:10.3389/fpls.2022.1040688)
Supplement: Supplementary file 12 [file Table_4.docx]

| Supplementary Table S4. Results of Bimolecular fluorescence complementation assays | | | | | | | | |
| --- | --- | --- | --- | --- | --- | --- | --- | --- |
|  | **NbToc34** | | | | | | | |
|  | **_Nt[GFP]_NbToc34** | **+/-** | **NbToc34_Nt[GFP]_** | **+/-** | **_Ct[GFP]_NbToc34** | **+/-** | **NbToc34_Ct[GFP]_** | **+/-** |
| Toc34 | _Ct[GFP]_NbToc34 | + | _Ct[GFP]_NbToc34 | + |  |  |  |  |
|  | NbToc34 _Ct[GFP]_ | + | NbToc34 _Ct[GFP]_ | + |  |  |  |  |
| TOC159  family | _Ct[GFP]_NbToc90 | - | _Ct[GFP]_NbToc90 | + | _Nt[GFP]_NbToc90 | - | _Nt[GFP]_NbToc90 | - |
|  | NbToc90_Ct[GFP]_ | - | NbToc90_Ct[GFP]_ | - | NbToc90_Nt[GFP]_ | - | NbToc90_Nt[GFP]_ | - |
|  | _Ct[GFP]_NbToc120 | - | _Ct[GFP]_NbToc120 | - | _Nt[GFP]_NbToc120 | - | _Nt[GFP]_NbToc120 | + |
|  | NbToc120_Ct[GFP]_ | - | NbToc120_Ct[GFP]_ | + | NbToc120_Nt[GFP]_ | - | NbToc120_Nt[GFP]_ | - |
|  | NbToc159A_Ct[GFP]_ | + | NbToc159A_Ct[GFP]_ | + | NbToc159A_Nt[GFP]_ | - | NbToc159A_Nt[GFP]_ | + |
|  | _Ct[GFP]_NbToc159A | + | _Ct[GFP]_NbToc159A | + | _Nt[GFP]_NbToc159A | - | _Nt[GFP]_NbToc159A | - |
|  | NbToc159B_Ct[GFP]_ | - | NbToc159B_Ct[GFP]_ | + | NbToc159B_Nt[GFP]_ | - | NbToc159B_Nt[GFP]_ | + |
|  | _Ct[GFP]_NbToc159B | + | _Ct[GFP]_NbToc159B | + | _Nt[GFP]_NbToc159B | - | _Nt[GFP]_NbToc159B | - |
|  | **NEGATIVE CONTROLS** | | | | | | | |
|  | **Nt[GFP]** | | | **+/-** | **Nt[GFP]** | | | **+/-** |
| Toc34 | _Ct[GFP]_NbToc34 | | | - | _Ct[GFP]_NbToc34 | | | - |
| TOC159  family | _Ct[GFP]_NbToc90 | | | - | _Ct[GFP]_NbToc90 | | | - |
|  | NbToc90_Ct[GFP]_ | | | - | NbToc90_Ct[GFP]_ | | | - |
|  | _Ct[GFP]_NbToc120 | | | - | _Ct[GFP]_NbToc120 | | | - |
|  | NbToc120_Ct[GFP]_ | | | - | NbToc120_Ct[GFP]_ | | | - |
|  | NbToc159A_Ct[GFP]_ | | | - | NbToc159A_Ct[GFP]_ | | | - |
|  | _Ct[GFP]_NbToc159A | | | - | _Ct[GFP]_NbToc159A | | | - |
|  | NbToc159B_Ct[GFP]_ | | | - | NbToc159B_Ct[GFP]_ | | | - |
|  | _Ct[GFP]_NbToc159B | | | - | _Ct[GFP]_NbToc159B | | | - |
